# Aqueous Humor Biomarkers, Efficacy, and Safety in Patients with Naïve Diabetic Macular Edema Treated with Faricimab: The ALTIMETER Study

**DOI:** 10.1016/j.xops.2026.101129

**Published:** 2026-02-26

**Authors:** Patricio G. Schlottmann, Amani A. Fawzi, Manuel Amador, Andreas Dieckmann, Kara Gibson, Carl Glittenberg, Chirag Jhaveri, Alex Kotak, Florie Mar, Ales Neubert, Veeral S. Sheth, Audrey Souverain, Bjoern Titz, Claudia Vazquez-Lombardi, Charles C. Wykoff, Focke Ziemssen, Stela Vujosevic

**Affiliations:** 1 Charles Centro Oftalmologico, Buenos Aires, Argentina; 2 Department of Ophthalmology, Feinberg School of Medicine, Northwestern University, Chicago, Illinois; 3 Genentech, Inc, South San Francisco, California; 4 F. Hoffmann-La Roche Ltd, Basel, Switzerland; 5 Roche Products Ltd, Welwyn Garden City, United Kingdom; 6 Retina Consultants of Austin and Austin Research Center for Retina, Austin, Texas; 7 Department of Ophthalmology and Visual Sciences, University of Illinois, Chicago, Illinois; 8 Retina Consultants of Texas, Houston, Texas; 9 Center of Ophthalmology, University of Leipzig, Leipzig, Germany; 10 Department of Biomedical, Surgical and Dental Sciences, University of Milan, Milan, Italy; 11 Eye Clinic, IRCCS MultiMedica, Milan, Italy

**Keywords:** Aqueous humor, Biomarkers, Diabetic macular edema, Faricimab, Multimodal imaging

## Abstract

**Objective:**

To explore associations between clinical endpoints, multimodal imaging, and aqueous humor (AH) biomarker patterns in patients with diabetic macular edema (DME) treated with faricimab.

**Design:**

A phase IIb, multicenter, open-label, single-arm, 24-week exploratory study.

**Participants:**

Adults with treatment-naïve DME, best-corrected visual acuity (BCVA) of 20–75 ETDRS letters, and central subfield thickness (CST) ≥325 μm were eligible to participate.

**Methods:**

Patients received six 4-weekly intravitreal doses of faricimab 6.0 mg. Functional and multimodal imaging assessments were conducted through week 24. Aqueous humor samples were collected at baseline and week 16 for targeted proteomic analysis. Patients were monitored for safety.

**Main Outcome Measures:**

Prespecified exploratory endpoints included changes from baseline over time in BCVA, CST, the proportion of patients with ≥2-step ETDRS Diabetic Retinopathy Severity Scale (DRSS) improvement, intraretinal/subretinal fluid and macular leakage on multimodal imaging, and AH biomarker patterns.

**Results:**

Ninety-nine patients were enrolled. At week 24, treatment with faricimab resulted in clinical and anatomical improvements consistent with phase III trials in DME (YOSEMITE/RHINE); the adjusted mean (95% confidence interval) change from baseline was +9.2 (7.5–10.9) letters for BCVA and –200.2 μm (–214.1 to –186.2) for CST, and 50.0% (37.2–62.8) of patients achieved ≥2-step DRSS improvement. Macular leakage area reduced from a median (interquartile range) of 28.6 mm^2^ (16.9–36.5) at baseline to 2.8 mm^2^ (0.9–15.3) at week 20. Total AH protein concentration decreased significantly from baseline to week 16 (*P* < 0.0001), with reductions across plasma-derived, inflammatory, and hypoxia response proteins. Notably, faricimab treatment significantly reduced the levels of key proteins associated with worse baseline DRSS scores (including angiopoietin-2, placental growth factor, and erythropoietin) and high baseline macular leakage, suggesting normalization of the AH proteomic profile. Safety was consistent with the known safety profile of faricimab.

**Conclusions:**

In patients with treatment-naïve DME, faricimab treatment resulted in meaningful improvements in functional and anatomical outcomes. These clinical gains were accompanied by a significant reduction in the levels of key AH proteins associated with disease severity, which underscores their potential as biomarkers for assessing disease activity and therapeutic response.

**Financial Disclosure(s):**

Proprietary or commercial disclosure may be found in the Footnotes and Disclosures at the end of this article.

Despite the advancements in anti-VEGF therapies, many patients with diabetic macular edema (DME) continue to experience suboptimal or unsustained outcomes despite treatment.^1^, ^2^, ^3^, ^4^, ^5^

The aim of developing novel therapies targeting new pathways is to address these limitations by offering additional efficacy and durability benefits. Faricimab, the first regulatory-approved intraocular bispecific antibody, inhibits VEGF-A and angiopoietin-2 (Ang-2), offering a dual mechanism that enhances vascular stability and reduces inflammation beyond the effects of VEGF inhibition alone.^6^^,^^7^ The Ang/Tie pathway (Tie referring to “tyrosine kinase with immunoglobulin and epidermal growth factor homology domains”) plays a key role in maintaining vascular stability, and its role in retinal disease has been described previously.^8^, ^9^, ^10^, ^11^ In brief, Ang-2 levels are elevated in pathologic conditions such as DME, disrupting the Ang/Tie pathway and resulting in vascular instability, increased inflammation, and enhanced VEGF-mediated angiogenesis.^8^, ^9^, ^10^, ^11^ The phase III faricimab studies in DME, the YOSEMITE (NCT03622580) and RHINE (NCT03622593) trials, demonstrated that faricimab led to greater and faster improvement of retinal drying and disease control compared with anti-VEGF treatment with aflibercept 2 mg alone, allowing for extended treatment intervals while preserving vision gains.^12^ At 2 years, the proportion of patients with ≥2-step ETDRS Diabetic Retinopathy Severity Scale (DRSS) improvement was greater in the YOSEMITE/RHINE faricimab every 8 weeks (Q8W) arm versus the aflibercept Q8W arm (pooled cohort).^13^

Several clinically relevant biomarkers are associated with improved treatment outcomes with faricimab, including reductions in intraretinal fluid (IRF), macular leakage, hyperreflective foci (HRF), epiretinal membranes, and central subfield thickness (CST).^13^, ^14^, ^15^, ^16^, ^17^, ^18^, ^19^ These biomarkers provide valuable insights into the added benefits of Ang-2 inhibition beyond VEGF inhibition, the efficacy of faricimab, and its potential to offer more comprehensive disease control through dual pathway inhibition.

Aqueous humor (AH) profiling has emerged as an innovative approach to further drive our understanding of disease mechanisms and treatment responses. It is considered a surrogate representative sample of the biological activity occurring in the vitreous body and retina, with protein concentrations in AH reflecting, to a certain extent, protein levels present in the vitreous and retina.^20^, ^21^, ^22^, ^23^, ^24^ By analyzing protein composition in the AH, it may be possible to identify potential biomarkers that correlate with clinical features and treatment outcomes. For instance, proteomic studies have shown that patients with DME exhibit elevated levels of specific inflammatory cytokines in their AH compared with healthy individuals,^25^^,^^26^ and the results of some studies suggest certain AH cytokines may act as biomarkers for response to treatment.^27^^,^^28^

Building on previous findings, the prospective ALTIMETER study (NCT04597918) was designed to explore associations between clinical endpoints, AH biomarker patterns, and multimodal imaging assessments. The prespecified endpoints included ETDRS best-corrected visual acuity (BCVA), ETDRS DRSS score (henceforth in the manuscript referred to as “DRSS”), CST, IRF and subretinal fluid (SRF), HRF, and macular leakage. Patients with treatment-naïve DME were treated with faricimab every 4 weeks (Q4W) for 6 months, with AH samples collected at baseline and at 16 weeks. Approximately 1000 proteins, including cytokines, chemokines, and growth factors, were analyzed in the AH using the proximity extension assay technology from Olink (Olink Proteomics AB). The aim of ALTIMETER was to generate insights into the pathophysiology of DME and the mechanisms of action of dual Ang-2 and VEGF-A inhibition with faricimab.

## Methods

### Ethics and Consent

ALTIMETER was conducted in accordance with the International Council for Harmonisation of Technical Requirements for Pharmaceuticals for Human Use (ICH) E6 Good Clinical Practice Guideline. The study adhered to the principles of the Declaration of Helsinki, the requirements of the ICH E2A guideline, US Food and Drug Administration regulations, and the European Union Clinical Trials Directive (2001/20/EC), as appropriate. ALTIMETER is registered at http://www.clinicaltrials.gov/ (NCT04597918). ALTIMETER was performed in adherence to all applicable local, state, and federal laws. Study protocols were approved by applicable institutional review boards and ethics committees before trial commencement, and all patients were required to provide informed consent to participate in the study prior to eligibility screening.

### Study Design and Participants

ALTIMETER was a phase IIb, multicenter, open-label, single-arm, 24-week exploratory study of the associations over time between clinical assessments, multimodal imaging assessments, and AH biomarker patterns in patients with DME treated with intravitreal (IVT) faricimab 6.0 mg Q4W. A full list of inclusion and exclusion criteria are provided in Table S1 (available at www.ophthalmologyscience.org).

• Key inclusion criteria:
  ○ Age ≥18 years;
  ○ Diagnosis of type 1 or 2 diabetes mellitus and regular use of insulin, other injectable, or oral antihyperglycemic agents for the treatment of diabetes mellitus;
  ○ Hemoglobin A1c ≤10%;
  ○ IVT treatment-naïve in study eye;
  ○ DME in study eye, defined per protocol as CST ≥325 μm (the average thickness between the internal limiting membrane [ILM] and Bruch membrane in the ETDRS grid) with SPECTRALIS (Heidelberg Engineering) spectral-domain OCT but converted by the central reading center (Wisconsin Reading Center, Merit CRO) to the ILM to retinal pigment epithelium (RPE) equivalent, which was ≥305 μm;
  ○ BCVA letter score of 20–75 letters on ETDRS-like charts (measured using the ETDRS visual acuity chart at a starting distance of 4 m);
  ○ Clear ocular media and adequate pupillary dilation to allow acquisition of good-quality retinal images to confirm diagnosis.
• Key exclusion criteria:
  ○ Currently untreated type 1 or 2 diabetes mellitus, or previously untreated patients who initiated oral or injectable antidiabetic medication within 3 months prior to day 1;
  ○ Presence of high-risk proliferative diabetic retinopathy (PDR; defined as DRSS >71 A) in study eye;
  ○ Any panretinal photocoagulation or macular laser photocoagulation treatment received in study eye;
  ○ Ocular disease other than DME that may confound assessment of the macula or affect central vision (e.g., age-related macular degeneration);
  ○ Any active or history of intraocular inflammatory disease (e.g., uveitis);
  ○ Prior treatment with periocular or IVT corticosteroids, or anti-VEGF in study eye; current treatment with brolucizumab or bevacizumab in nonstudy eye, or prior treatment with corticosteroid implant in nonstudy eye;
  ○ Any intraocular surgery (e.g., cataract surgery) within 3 months prior to day 1 or any planned surgery during the study (1 rescreening for this criterion was permitted);
  ○ Uncontrolled glaucoma in study eye, surgery for glaucoma or laser procedure prior to the screening visit, or iris surgery/laser within prior 6 months;
  ○ Nonfunctioning nonstudy eye.

If both eyes were considered eligible for inclusion in the study, the eye with worse BCVA at screening was selected; however, the other eye could be selected at investigator discretion if it was deemed more suitable for study.

### Schedule of Assessments

Patients included in the study were assessed on day 1 (baseline) and again in the clinic at weeks 4, 8, 12, 16, 20, and 24 (patients who discontinued study treatment returned for an early termination visit ≥28 days and <35 days after their last study treatment). Visual acuity assessment (BCVA, assessed on ETDRS chart) was conducted at every visit.

### Study Drug Administration

Patients received a total of 6 doses of faricimab 6.0 mg, which were administered by an IVT injection Q4W, starting on day 1 and ending at week 20.

### Ocular Assessments

• Every visit: Ultra-widefield (UWF) color fundus photographs; spectral-domain OCT and (if capable) swept-source OCT, swept-source OCT-angiography (if capable), otherwise spectral-domain OCT-angiography;
• Screening, baseline, and week 20: stereo color fundus photographs with 7-modified field imaging;
• Screening and week 20: UWF fundus fluorescein angiography (FFA);
  o UWF-FFA images were taken at 15 to 45 seconds (4–6 images), 2 minutes (3 images), 5 minutes (3 images), and 10 minutes (3 images) after injection of fluorescein.

### Safety Assessments

Patients underwent comprehensive safety monitoring during the study and were assessed for adverse events (AEs) at each treatment visit by the investigator. Intraocular pressure was measured pretreatment and posttreatment (within 30 minutes ± 15 minutes) at every visit. Patients returned for a safety follow-up visit 4 weeks (between 28 and 35 days) after their final study treatment.

### Grading and Assessment of Images

Ocular images (OCT, color fundus photographs, and FFA) were independently assessed by masked evaluators at the central reading center (Wisconsin Reading Center). Images were assessed for retinal thickness, absence of DME, presence of SRF and IRF, and area of leakage (fluorescein leakage within ETDRS grid on FFA), and graded for DRSS. The mid-late phase UWF-FFA images were used to evaluate leakage, atrophy, and abnormal vessels; leakage was assessed by reading center–certified graders using Optos scanning ophthalmoscope technology (V2 Vantage software version 3.4 or higher, or Optos Advance software version 4.2 or higher).

Spectral-domain OCT volume scans were segmented using a deep learning–based algorithm (developed using B-scans from the BOULEVARD clinical trial [NCT02699450]) to detect IRF, SRF, and HRF (upper size limit for HRF was ≤50 μm).^19^^,^^29^^,^^30^ For IRF, SRF, and HRF, the total volume was assessed; for HRF, the object count was also assessed.

### AH Biomarker Analysis

Mandatory AH taps (>90 μL) from the study eye were collected on day 1 (baseline; just prior to faricimab injection #1) and at week 16 (just prior to faricimab injection #5). Aqueous humor samples (25 μL each) were shipped on dry ice to Olink Proteomics AB and analyzed using the Olink Target 96 platform. Each sample was measured once. The following 12 panels were assessed: cardiometabolic, cell regulation, cardiovascular II, cardiovascular III, development, immune response, inflammation, metabolism, neurology, oncology II, oncology III, and organ damage. A comprehensive list of the proteins included in these panels is provided in Table S2 (available at www.ophthalmologyscience.org). Quality control for the Olink proteomics data included assessments of detectability, plate effects, and outlier identification. Protein levels were quantified on a relative scale (arbitrary log2 scale unit) and expressed as normalized protein expression (NPX). While the Olink platform provides relative quantification (NPX) rather than absolute concentration, its technical performance has been extensively validated. Independent evaluations have demonstrated high precision (median intraplate/interplate coefficient of variation <15%) and specificity, attributed to the dual-recognition mechanism of this proximity extension assay.^31^ Furthermore, the assay's suitability for AH profiling has previously been confirmed; with the platform demonstrating high detection rates (>700 proteins) and sufficient sensitivity to detect nuanced biological variances driven by ocular confounders such as topical medication or lens status.^22^^,^^23^

Total AH protein concentrations were determined using the NanoOrange Protein Quantitation Kit (#N-6666; Thermo Fisher Scientific) according to the manufacturer's instructions.

For functional interpretation of DME/leakage-related effects, protein responses within selected biological categories were evaluated. These categories included plasma proteins with the 50 highest concentrations according to the Human Protein Atlas database (www.proteinatlas.org),^32^ proteins related to Ang-2 and VEGF-A, and the following Gene Ontology (GO) terms: “cytokine activity” (GO:0005125), “inflammatory response” (GO:0006954), “sprouting angiogenesis” (GO:0002040), “angiogenesis” (GO:0001525), “VEGF signaling pathway” (GO:0038084), and “response to hypoxia” (GO:0001666).

To illustrate individual protein responses, we selected representative proteins across these categories with relevance to the pathology of the disease: Ang-2 (core regulator and target), VEGF-A (core regulator and target), ICAM-1 (intracellular adhesion molecule 1; endothelial activation/inflammation marker),^33^ selectin-E (endothelial activation/inflammation marker),^34^ ESM-1 (endothelial cell-specific molecule; endothelial function/dysfunction marker),^35^ CCL-19 (C-C motif chemokine ligand 19; immune cell infiltration/inflammation marker),^35^ and FCN-2 (ficolin-2; plasma protein and candidate leakage marker).^36^

### Exploratory Endpoints

The ALTIMETER study did not have a primary endpoint as it was designed to explore multiple endpoints with the objective of generating hypotheses and to provide a comprehensive understanding of DME disease mechanisms and responses to treatment with faricimab. The following exploratory endpoints are reported in this paper:

• Changes from baseline in BCVA as measured on the ETDRS chart over time;
• Proportion of patients with ≥2-step improvement in DRSS over time;
• Changes from baseline in IRF over time;
• Changes from baseline in SRF over time;
• Changes from baseline in CST over time;
• Time to first absence of DME (CST <305 μm [ILM-RPE]);
• Changes from baseline in multimodal imaging over time, including CST, IRF, SRF, HRF, and macular leakage at baseline and week 20;
• Changes from baseline in AH biomarker patterns.

A full list of other/all exploratory endpoints is included in Table S3 (available at www.ophthalmologyscience.org).

### Statistical Methods

All exploratory analyses were conducted in the modified intent-to-treat population. The modified intent-to-treat population comprised all patients enrolled in the study who received any dose of faricimab. The safety population consisted of all patients who received ≥1 dose of faricimab. All AEs were coded to the preferred term using the Medical Dictionary for Regulatory Affairs version 25.1.^37^ Baseline was defined as the last available measurement obtained on or prior to the day of the first dose of faricimab. For the continuous variables, descriptive statistics are reported, and for categorical variables, number and percentage are reported. Best-corrected visual acuity and CST outcomes were analyzed using a mixed model for repeated measures, which was adjusted for visit, age, region, and baseline BCVA or CST (as appropriate). Patients and visits were treated as factor variables, and an unstructured covariance was used to model the within-patient errors. Kaplan–Meier estimates were generated for time to first absence of DME through week 24.

Statistical tests were 2-sided, and the statistical significance level was 5%. As this was an exploratory study, no adjustments to the type 1 error rate for multiplicity were applied, except for the Olink measurements.

To evaluate the protein profiles measured by Olink technology, we excluded proteins detected in <30% of samples with NPX values above the limit of detection. However, analytes with significant differential detectability in any disease group were retained, even if detected in <30% of samples (Fisher exact test, false discovery rate [FDR] <0.01). For the remaining proteins, NPX values below the limit of detection were retained as the best available estimates of their abundances. Differential abundance between conditions was assessed using linear models with the limma package in R (R Foundation for Statistical Computing). Continuous outcomes were dichotomized using their medians, and covariates were included for specific comparison. All statistical analyses of Olink data were adjusted for lens status due to the general influence of lens status on AH protein levels.^22^ Moderated t-tests were performed using the empirical Bayes method, and the resulting *P* values were adjusted for multiple testing using the Benjamini–Hochberg procedure to control the FDR. Analytes with an adjusted *P* value <0.05 were considered differentially abundant.

## Results

### Patient Disposition

In total, 99 patients were enrolled in the study across 23 study sites in 7 countries; 90 patients (90.9%) completed study treatment. The reasons for treatment discontinuation were withdrawal by patient (n = 6, 6.1%), loss to follow-up (n = 2, 2.0%), and death (n = 1, 1.0%).

### Participant Demographics and Baseline Characteristics

Demographic, ocular, and nonocular characteristics at baseline of patients included in the study are summarized in Table 1.

**Table 1:** Baseline Patient Characteristics (Study Eye), mITT Population

Characteristic	Faricimab 6.0 mg Q4W (N = 99)
Age, yrs, mean (SD)	59.5 (9.8)
Male, n (%)	61 (61.6)
Race	
White, n (%)	86 (86.9)
Ethnicity, n (%)	
Not Hispanic or Latino	73 (73.7)
Hispanic/Latino	25 (25.3)
Unknown	1 (1.0)
Geographical region, n (%)	
USA and Canada	62 (62.6)
Rest of the world	37 (37.4)
BCVA, ETDRS letters, mean (SD)	62.5 (11.8)
Lens status	
Phakic	77 (77.8)
Pseudophakic	20 (20.2)
Aphakic	2 (2.0)
CST (ILM-RPE), μm, mean (SD)	464 (149.5)
Median (min–max)	426.0 (292.0–1123)
Presence of macular leakage, n/N (%)	97/97 (100)
IRF present, n (%)∗	98 (99)
IRF volume,† nL, mean (SD), central 3 mm	482.4 (368.3)
Median (min–max)	384.3 (8.6–1882.4)
SRF present,∗ n (%)	37 (37.4)
SRF volume,† nL, mean (SD), central 3 mm	55.0 (148.8)
Median (min–max)	0.0 (0.0–862.0)
Global DR status, n (%)	
DR questionable (ETDRS-DRSS levels 14 A–Z, 35 E–F)	1 (1.0)
Mild/moderate NPDR (ETDRS-DRSS levels 35 A–F/43 A–B)	48 (48.5)
Moderately severe/severe NPDR (ETDRS-DRSS levels 47 A–D/53 A–E)	31 (31.3)
PDR (ETDRS-DRSS levels ≥61)	11 (11.1)
Cannot grade (ETDRS-DRSS level 90)	8 (8.1)

BCVA = best-corrected visual acuity; CST = central subfield thickness; DR = diabetic retinopathy; DRSS = Diabetic Retinopathy Severity Scale; ILM = internal limiting membrane; IRF = intraretinal fluid; mITT = modified intent-to-treat; NPDR = nonproliferative diabetic retinopathy; PDR = proliferative diabetic retinopathy; Q4W = every 4 weeks; RPE = retinal pigment epithelium; SD = standard deviation; SRF = subretinal fluid.

∗ Presence of IRF and SRF was based on reading center gradings.

† Intraretinal fluid and SRF volumes were segmented and measured using a deep learning–based algorithm.

### Efficacy Results

All results reported are for the modified intent-to-treat population, unless stated otherwise.

#### Vision and Anatomy

**Figure 1:**
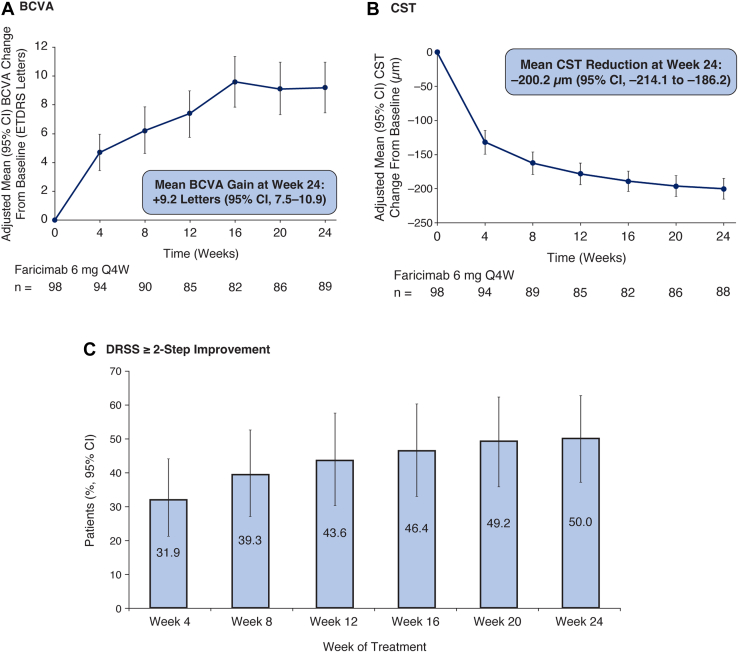
Key anatomic and functional outcomes (mITT population^a^). **A,** Best-corrected visual acuity change from baseline, **(B)** CST change from baseline (mITT population), and **(C)** improvement of ≥2 steps from baseline in ETDRS DRSS. ^a^mITT population: all patients enrolled in ALTIMETER who received any dose of faricimab. BCVA = best-corrected visual acuity; CI = confidence interval; CST = central subfield thickness; DRSS = Diabetic Retinopathy Severity Scale; mITT = modified intent-to-treat; Q4W = every 4 weeks.

Robust vision gains and reductions in CST were observed over time during treatment with faricimab 6.0 mg Q4W (Fig 1A, B). The adjusted mean change from baseline in BCVA at end of study (week 24) was +9.2 letters (95% confidence interval [CI], 7.5–10.9). The adjusted mean change from baseline in CST at week 24 was –200.2 μm (95% CI, –214.1 to –186.2). The median time to first absence of DME (CST [ILM-RPE] <305 μm) was 8.0 weeks (95% CI, 8.0–12.0). At end of study (week 24), the cumulative incidence of first absence of DME was 81.6% (80 of 98 patients) (Fig S1, available at www.ophthalmologyscience.org). The proportion of patients with ≥2-step improvement in DRSS from baseline increased steadily over time and reached 50.0% at week 24 (95% CI, 37.2–62.8; Fig 1C).

#### Fluid

**Figure 2:**
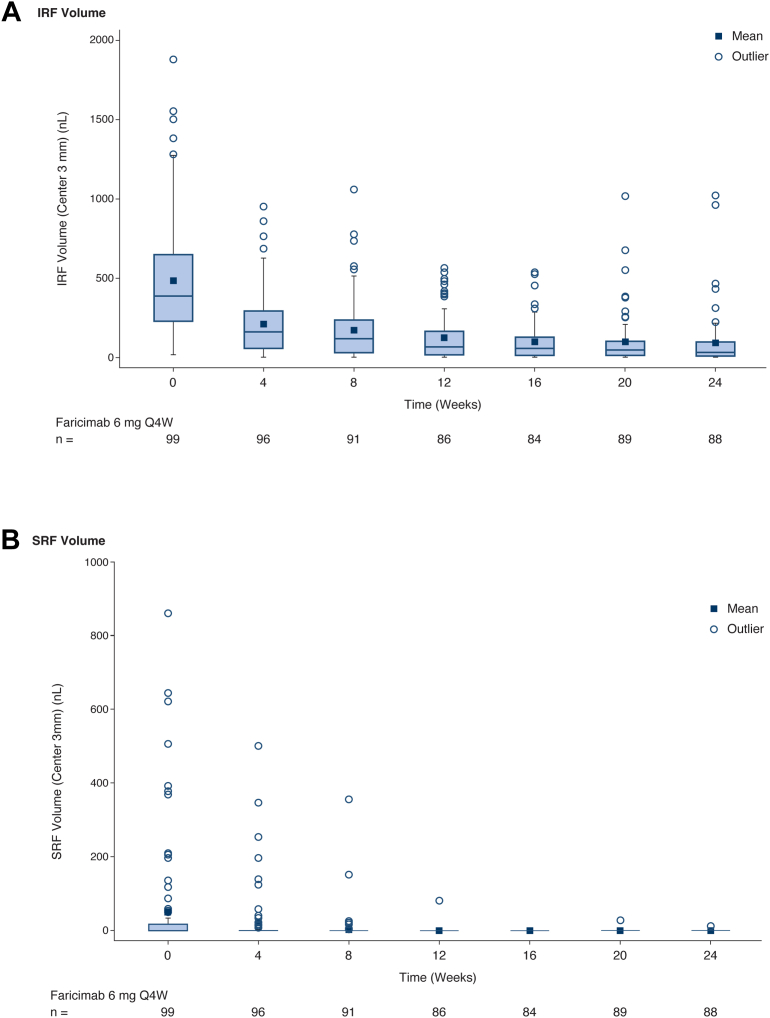

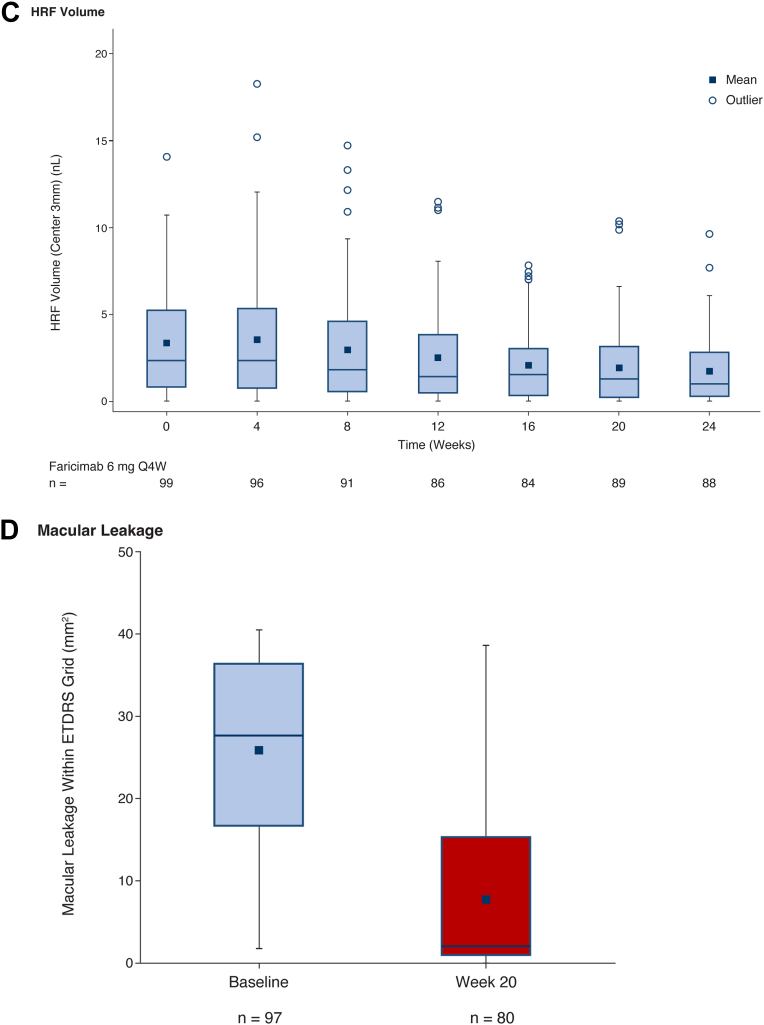
**A,** Intraretinal fluid volume, **(B)** SRF volume, and **(C)** HRF volume (ILM to RPE) in the center 3 mm of the ETDRS grid, and **(D)** macular leakage area (UWF-FFA assessment of fluorescein leakage) in the ETDRS grid over time; absolute values in the mITT population. In each figure, the box represents the IQR, and the horizontal line within the box is the median; in parts **A**, **B**, and **C**, the vertical lines show the range between minimum and maximum observations within lower fences, and the circles show outliers. In part **D**, the vertical lines show the range of minimum to maximum values. HRF = hyperreflective foci; ILM = internal limiting membrane; IQR = interquartile range; IRF = intraretinal fluid; mITT = modified intent-to-treat; Q4W = every 4 weeks; RPE = retinal pigment epithelium; SRF = subretinal fluid; UWF-FFA = ultra-widefield fundus fluorescein angiography.

At baseline, almost all patients had IRF present; only 1 of 99 patients (∼1%) had IRF absence (defined as IRF absent or outside of the central subfield only). The proportion of patients with IRF absence at week 24 was 23 of 88 patients (26.1% [95% CI, 17.3–36.6]). The proportions of patients with SRF absence in the central subfield at baseline and at week 24, respectively, were 41 of 61 (67.2%) and 54 of 55 (98.2%). The absolute values of IRF volume and SRF volume over time, both measured in the ETDRS grid (3 mm diameter), are shown in Figure 2A, B, respectively. The absolute IRF volume (median) at baseline was 384.3 nL (interquartile range [IQR]: 226.2–646.9), reducing to 26.8 nL (IQR: 6.6–89.9) at week 24, a median reduction of 343.3 nL compared with baseline. For SRF, the median absolute volumes at baseline and at week 24 were 0.0 nL (IQR: 0.0–19.2) and 0.0 nL (IQR: 0.0–0.0), respectively.

#### HRF and Macular Leakage

The absolute value of HRF volume (ILM-RPE; center 3 mm) is shown in Figure 2C. The change in HRF count over time is shown in Figure S2 (available at www.ophthalmologyscience.org). Mean HRF volume (standard deviation) was 3.3 nL (3.1) at baseline and 1.8 nL (1.9) at week 24 (mean change from baseline –1.5 [2.3]); median HRF volume was 2.3 nL (IQR: 0.8–5.3) at baseline and 1.0 nL (IQR: 0.3–2.8) at week 24. Mean absolute HRF count (standard deviation) was 103.3 (90.8) at baseline and 56.7 (59.6) at week 24 (mean change from baseline: –44.8 [67.6]); median HRF count reduced from 71.0 (IQR: 29.0–162.0) at baseline to 33.0 (IQR: 13.0–90.5) at week 24 (median change from baseline: –25.5 [IQR: –70.5 to –7.5]). With respect to macular leakage within the ETDRS grid, the median area at baseline (UWF-FFA) was 28.6 mm^2^ (IQR: 16.9–36.5) and at week 20 was 2.8 mm^2^ (IQR: 0.9–15.3; Fig 2D).

### AH Biomarker Analyses

#### Total AH Protein Concentration and Protein Response Profiles

**Figure 3:**
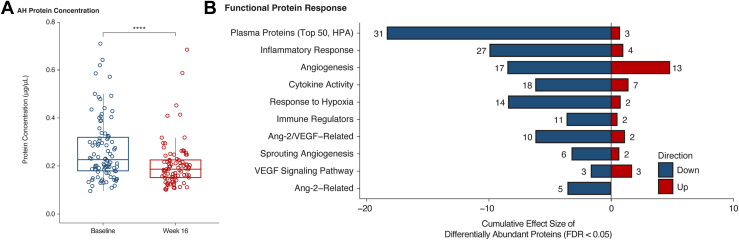
Pharmacodynamic effect of faricimab treatment on AH protein profile, mITT population. **A,** Aqueous humor protein concentration measured by a total protein assay. The boxplot displays the distribution of protein concentrations with overlaid individual data points. The comparison between baseline and week 16 was performed using a linear fixed effects model for the log2-transformed protein concentrations, adjusting for lens status as a covariate and including the subject as a fixed effect to account for intraindividual differences. Contrasts were estimated using estimated marginal means (∗∗∗∗*P* < 0.0001). **B,** Functional protein response, week 16 compared with baseline: cumulative effect size of differentially abundant proteins quantified by the Olink approach, grouped into selected functional categories. For each category, the bar plot shows the sum of the log2-fold change of significantly upregulated and downregulated proteins when comparing week 16 to baseline (FDR <0.05). The number of significantly upregulated and downregulated proteins is indicated in the text labels. AH = aqueous humor; Ang-2 = angiopoietin-2; FDR = false discovery rate; HPA = Human Protein Atlas; mITT = modified intent-to-treat.

The total protein concentration in AH (mean ± standard deviation) was significantly lower (*P* < 0.0001) at week 16 (0.20 ± 0.10 μg/μL) compared with baseline (0.27 ± 0.13 μg/μL) (Fig 3A).

Using Olink technology, 708 proteins were quantified across the AH samples after quality control. Differential abundance analysis identified 140 proteins with significantly increased levels and 193 proteins with significantly decreased levels at week 16 compared with baseline (FDR-adjusted *P* < 0.05) (Fig S3, available at www.ophthalmologyscience.org). Out of the 50 differentially abundant proteins with the largest fold changes, the levels of 48 proteins were significantly decreased, whereas levels of 2 proteins were significantly upregulated at week 16 compared with baseline (Fig S4A, available at www.ophthalmologyscience.org).

When assessing these protein responses to faricimab treatment across functional categories relevant to DME biology, a general reduction was observed in most categories (Fig 3B). Specifically, for proteins likely originating from plasma but detected in AH (considered candidate markers of leakage), there was a decrease in the expression of 31 proteins and an increase in the expression of 3 proteins at week 16. Of proteins involved in the inflammatory response, there was a reduction in the expression of 27 proteins and an increase in the expression of 4 proteins. Hypoxia response proteins included 14 downregulated proteins and 2 upregulated proteins. Of the Ang-2/VEGF-related proteins, 10 were downregulated, including VEGF-A and Ang-2, and 2 proteins were upregulated. Notably, angiogenesis-related proteins presented a more balanced response, with 17 downregulated proteins and 13 upregulated proteins, and VEGF signaling pathway proteins showed an equal number of upregulated and downregulated proteins (3 down, 3 up) at week 16 compared with baseline. Detailed individual protein responses by category are illustrated in Figure S4B.

#### Associations of AH with Clinical Features and Imaging Biomarkers at Baseline

To assess the relationship between the AH protein profile and anatomical and functional parameters at baseline, an additional differential abundance analysis was conducted (Fig S5, available at www.ophthalmologyscience.org). A substantial number of proteins significantly associated with various parameters (FDR-adjusted *P* < 0.05; lens status adjusted) were identified. These proteins predominantly showed positive associations (i.e., proteins were detected at higher concentrations in the presence of parameter values higher than the median parameter value). Specifically, at baseline, AH protein associations with macular leakage area (431 positive, 11 negative), IRF volume (180 positive, 3 negative), CST (54 positive, 0 negative), and DRSS (7 positive, 0 negative; PDR vs. mild/moderate nonproliferative diabetic retinopathy [NPDR]) were found. No significant associations were observed for SRF volume, HRF counts, and BCVA (Fig S5A).

The extensive number of proteins linked to macular leakage suggested that this analysis revealed general patterns rather than specific associations for individual proteins. Therefore, for a more precise identification of associations, these evaluations were adjusted for total AH protein concentration. This analysis identified proteins specifically associated with macular leakage (76 positive, 1 negative) and DRSS (3 positive, 0 negative) at baseline (Fig S5A right-hand panel). The 77 proteins specifically associated with macular leakage at baseline after protein concentration adjustment spanned all previously discussed categories and included Ang-2/VEGF-related proteins such as Ang-2, VEGF-A, placental growth factor (PlGF), VEGF-D, CDH-5, ICAM-1, and PDGF-A (Fig S5B). The 3 proteins specifically associated with DRSS (PDR vs. mild/moderate NPDR) at baseline after protein concentration adjustment were Ang-2, erythropoietin (EPO), and PlGF.

#### Differential AH Protein Response to Faricimab Based on Baseline Macular Leakage and DRSS

**Figure 4:**
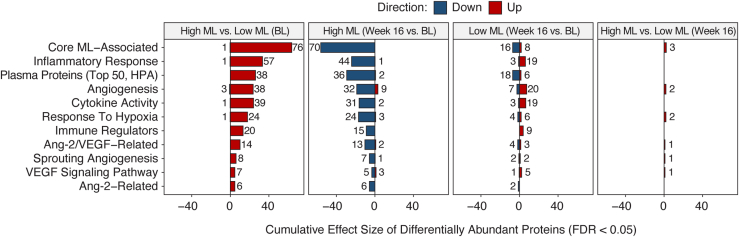
Aqueous humor protein profile patterns for patients with high vs. low ML at BL. Cumulative effect size of differentially abundant proteins, comparing treatment and BL ML groups, grouped into selected functional categories. For each category, the bar plot displays the sum of the log2 fold change of significantly upregulated and downregulated proteins for the indicated comparison. The number of significantly upregulated and downregulated proteins is provided in the text labels. AH = aqueous humor; Ang-2 = angiopoietin-2; BL = baseline; FDR = false discovery rate; HPA = Human Protein Atlas; ML = macular leakage.

To further investigate the link between AH protein responses and macular leakage, patients with high and low macular leakage at baseline (Fig 4) were compared. Eyes with high baseline macular leakage (defined as those with macular leakage above the median value at baseline) showed elevated protein levels and distinct profiles compared with those with low macular leakage (defined as eyes with macular leakage below the median value at baseline). For instance, at baseline, 57 inflammatory response proteins were elevated in eyes with high macular leakage, and the levels of 44 of these proteins reduced in the high leakage group after 16 weeks of faricimab treatment. Similarly, the levels of 20 immune regulation proteins were elevated at baseline, and there was a reduction in the levels of 15 of these proteins after faricimab treatment. The levels of 6 Ang-2-related proteins were also higher in eyes with high macular leakage, all of which decreased by week 16. In contrast, eyes with low macular leakage at baseline showed less change in protein expression at week 16, indicating a generally lower fold-change response to faricimab treatment.

At week 16 posttreatment, the protein profiles of eyes that initially had high macular leakage became more similar to those of eyes with low macular leakage at baseline. This indicates a comprehensive “normalization” of protein levels after faricimab treatment, removing the previously observed protein differences between eyes with different baseline macular leakage values. This is further illustrated in the response to treatment of AH proteins grouped by functional category: the levels were “renormalized” at week 16 after treatment with faricimab (Fig 4). Similar patterns of “renormalization” at week 16 after treatment with faricimab were shown for Ang-2, CCL-19, ESM-1, FCN-2, ICAM-1, selectin-E, and VEGF-A, as representative proteins of the vascular stability/inflammation interface (see Methods, AH Biomarker Analysis for details) (Fig S6, available at www.ophthalmologyscience.org).

**Figure 5:**
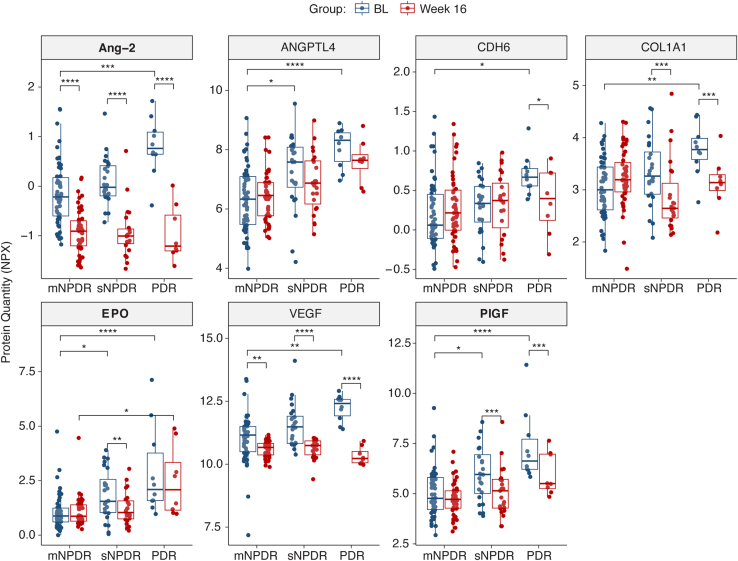
Aqueous humor protein responses for proteins associated with DRSS at BL. Box plots show unadjusted protein quantities (NPX values) for proteins associated with DRSS at baseline. Diabetic Retinopathy Severity Scale categories are: mNPDR, mild/moderate NPDR; sNPDR, moderately severe/severe NPDR; and PDR. Proteins labeled in bold (Ang-2, EPO, and PlGF) were significantly associated with DRSS at baseline also after total protein concentration adjustment. Individual data points and statistical significance markers are included (∗FDR <0.05, ∗∗FDR <0.01, ∗∗∗FDR <0.001, ∗∗∗∗FDR <0.0001). Ang-2 = angiopoietin-2; ANGPTL4 = angiopoietin-like 4; BL = baseline; CDH6 = cadherin-6; COL1A1 = collagen type 1 alpha-1; DRSS = Diabetic Retinopathy Severity Scale; EPO = erythropoietin; FDR = false discovery rate; m = moderate; NPDR = nonproliferative diabetic retinopathy; NPX = normalized protein expression; PDR = proliferative diabetic retinopathy; PlGF = placental growth factor; s = moderately severe/severe.

The responses of the 7 AH proteins associated with DRSS at baseline are illustrated in Figure 5. Of note, among these proteins, the associations of Ang-2, EPO, and PlGF remained significant (FDR <0.05) after adjusting for protein concentration. Angiopoietin-2, as a direct target of faricimab, demonstrated the most pronounced and significant reduction in detectable levels at week 16 compared with baseline across all evaluated DRSS categories (FDR <0.05). Placental growth factor levels were significantly (FDR <0.05) reduced with faricimab treatment at week 16 compared with baseline in eyes with moderately severe/severe NPDR and PDR. Erythropoietin levels were significantly reduced with faricimab treatment at week 16 compared with baseline only in eyes with moderately severe/severe NPDR.

### Safety

Safety outcomes were consistent with the established profile of faricimab in the phase III studies^12^^,^^13^ as well as observational studies in a clincal setting;^38^^,^^39^ results are summarized in Table 2. There were no AEs that resulted in study treatment discontinuation. Of the patients, 23.2% were reported to have ocular AEs; only 1 of these (conjunctival hemorrhage in 1 patient) was treatment related, and none of the ocular AEs were deemed serious AEs. There were no intraocular inflammation events (including retinal vasculitis or retinal occlusive vasculitis) and no cases of endophthalmitis or retinal vascular occlusions. There were no ocular AEs of special interest in the study eye.

**Table 2:** Safety Outcomes for the Safety Population∗

Summary of AEs, n (%)	Faricimab 6 mg Q4W (N = 99)
Patients with ≥1 AE (ocular or nonocular)	45 (45.5)
Patients with ≥1 SAE	8 (8.1)
Total number of deaths	1 (1.0)
Patients withdrawn from study due to an AE	1 (1.0)
Patients withdrawn from study treatment due to an AE	0
Patients with ≥1 AESI	1 (1.0)†
Patients with ≥1 ocular AE in study eye	23 (23.2)
SAE	0
AE leading to treatment withdrawal	0
Treatment-related AE	1 (1.0)
Intraocular inflammation‡	0
Retinal vascular occlusion§	0
Ocular AESI||	0
Infectious endophthalmitis¶	0
Rhegmatogenous retinal detachment	0
Retinal tear	0
Nonocular arterial thrombotic event or cerebrovascular hemorrhagic event	2 (2.0)

AE = adverse event; AESI = adverse event of special interest; Q4W = every 4 weeks; SAE = serious adverse event.

∗ Defined as all patients enrolled in ALTIMETER who received any amount of study treatment.

† Ocular AESI in the fellow eye.

‡ Intraocular inflammation events include anterior chamber flare, anterior chamber inflammation, chorioretinitis, choroiditis, cyclitis, eye inflammation, iridocyclitis, iritis, keratic precipitates, keratouveitis, noninfective chorioretinitis, noninfectious endophthalmitis, ocular vasculitis, postprocedural inflammation, retinal vasculitis, retinal occlusive vasculitis, uveitis, and vitritis.

§ Retinal vascular occlusion events include arterial occlusive disease, retinal artery embolism, retinal artery occlusion, retinal vein occlusion, and venous occlusion.

|| AESIs are defined as drop in visual acuity score of ≥30 letters, associated with severe intraocular inflammation, intervention required to prevent permanent vision loss (compared with the assessment of visual acuity immediately prior to the most recent assessment) lasting ≥1 hour; requires surgical or medical intervention to prevent permanent loss of sight; or is associated with severe intraocular inflammation; and suspected transmission of infectious agent by study drug.

¶ Infectious endophthalmitis events include endophthalmitis, *Candida* endophthalmitis, mycotic endophthalmitis, pseudo-endophthalmitis, bacterial endophthalmitis, and panophthalmitis.

## Discussion

ALTIMETER confirmed the robust efficacy and safety of faricimab, a dual Ang-2/VEGF-A inhibitor, previously demonstrated in the YOSEMITE and RHINE phase III trials and extended these findings by comprehensively evaluating AH biomarkers. In YOSEMITE (N = 940) and RHINE (N = 951), faricimab 6.0 mg (Q8W or treat-and-extend [T&E] up to every 16 weeks dosing) demonstrated comparable vision gains and improved anatomical outcomes compared with aflibercept 2.0 mg Q8W.^12^^,^^13^ At year 1, adjusted mean BCVA change from baseline was comparable with faricimab Q8W +11.2 letters, faricimab T&E +11.2 letters, and aflibercept 2.0 mg Q8W +10.5 letters.^40^ Best-corrected visual acuity improvements were maintained at year 2 (+10.8, +10.4, and +10.3 letters, respectively).^13^ Greater CST reductions were achieved with faricimab at year 1 (–200.9 μm and –192.4 μm versus –170.2 μm) and similarly at year 2 (–209.4 μm, –201.0 μm versus –190.9 μm).^13^^,^^40^ Higher rates of IRF resolution by year 2 were also achieved with faricimab (44%–63%) compared with the aflibercept arm (36%–41%).^13^ Furthermore, >60% of patients in the faricimab T&E arm achieved every 16 weeks dosing by year 2, demonstrating extended durability. Faricimab was well tolerated through 2 years with a safety profile comparable to aflibercept, and no cases of retinal vasculitis or occlusive retinal vasculitis were reported.^13^

Consistent with these pivotal results, treatment with faricimab in ALTIMETER led to improvements across functional outcomes (BCVA), anatomical measures (CST), and key morphological biomarkers (retinal fluid, HRF, and macular leakage). Notably, >50% of patients in ALTIMETER achieved ≥2-step improvement in DRSS by week 24. This substantial response under a Q4W dosing regimen aligns with the week 96 results from YOSEMITE and RHINE, where 52% and 44% in the faricimab Q8W and T&E groups achieved this improvement compared with 43% in the aflibercept group. The safety profile of faricimab in ALTIMETER was consistent with that observed in the YOSEMITE and RHINE studies.^12^

A key morphological finding was the substantial reduction of IRF volume, where the median volume decreased from 384.3 nL at baseline to 26.8 nL at week 24. This aligns with the YOSEMITE/RHINE outcomes, in which faricimab consistently led to an increased proportion of patients with IRF resolution compared with aflibercept 2 mg treatment.^12^^,^^17^ This is clinically critical, as the presence of IRF has been shown to have a negative impact on vision, and its early reduction is associated with improved visual outcomes in patients with DME.^30^^,^^41^ Furthermore, the magnitude of the reduction in macular leakage after treatment with faricimab in ALTIMETER was consistent with that recorded in the YOSEMITE and RHINE trials, in which faricimab had a stronger effect than aflibercept 2 mg.^14^ The consistency of these findings indicates a role for Ang-2 inhibition and suggests that dual Ang-2/VEGF inhibition provides improved disease control and vascular stability versus VEGF inhibition alone.

The ALTIMETER study was uniquely designed to further investigate the molecular basis for enhanced vascular stability, incorporating mandatory AH sampling that enabled comprehensive analysis of the proteomic changes associated with disease activity and treatment response. Faricimab demonstrated a strong pharmacodynamic effect, evidenced by a significant reduction in total AH protein concentration at week 16. This was further substantiated by proteomic profiling, which identified significant reductions (FDR <0.05) in 31 of the 50 highest-concentrated plasma-derived proteins. This marked decrease in the levels of plasma-derived proteins is consistent with reduced vascular leakage, indicating the substantial stabilizing effect of faricimab on the vasculature. In addition to its effects on vascular stability, AH protein profiling revealed that faricimab significantly decreased the levels of key inflammatory and immune-regulating proteins, such as chemokines.

The extensive associations between AH protein profiles and anatomical parameters at baseline underscore the complex interplay between protein expression and disease pathology in DME and diabetic retinopathy.^42^^,^^43^ Significant associations with macular leakage, IRF, CST, and DRSS categories suggest that AH protein levels reflect underlying disease processes. However, the large number of significantly associated proteins (e.g., 442 proteins associated with macular leakage) suggests that some of these associations might lack specificity, making it challenging to derive clear biological conclusions. Therefore, to pinpoint the most relevant proteins, we evaluated the associations after adjusting for total AH protein concentration. This refined analysis revealed that, at baseline, higher levels of several key proteins, including Ang-2, EPO, and PlGF, were significantly and positively correlated with worse DRSS scores, directly linking these proteins to more severe disease ahead of treatment.

The therapeutic relevance of these baseline protein associations was confirmed by the effect of faricimab on the key proteins linked to DRSS severity. Treatment led to significant reductions in Ang-2, EPO, and PlGF. The pronounced decline in the direct target, Ang-2, across all DRSS categories confirms target engagement and reinforces the therapeutic importance of modulating the Ang/Tie pathway in this disease. Interestingly, in addition to its broad inhibition of VEGF-A, faricimab also normalized PlGF levels in patients with more advanced disease. This effect is particularly noteworthy because PlGF is a direct target of other approved therapeutics, namely aflibercept. The ability of faricimab to potently suppress multiple, distinct proteins that are tightly correlated with clinical measures of severity provides a strong molecular rationale for its comprehensive efficacy and underscores the potential of these proteins as biomarkers.

Analyzing the AH protein response in eyes stratified by the level of macular leakage at baseline provided further insights into the therapeutic impact of faricimab. Eyes with high macular leakage had elevated levels of inflammatory, immune regulatory, and Ang-2 related proteins at baseline compared with the low macular leakage group. Faricimab treatment led to significant reductions in these proteins, particularly in the high leakage group, effectively “normalizing” the protein profile toward that of the low leakage group. This normalization supports the hypothesis that dual inhibition of VEGF-A and Ang-2 can effectively modulate both the vascular permeability and immune dysregulation characteristics of more severe disease. This molecular rebalancing promotes a more homeostatic intraocular environment. Although baseline biomarkers were not directly associated with visual acuity, the modulation of the AH protein profile over time provides a molecular rationale for the overall clinical improvements. Specifically, the “normalization” of the protein profile at week 16, driven by reductions in vascular permeability factors and inflammatory mediators, aligns with the robust resolution of macular leakage and CST reduction. This restoration of vascular stability likely contributes to the meaningful improvements in retinal anatomy and vision.

From a clinical perspective, AH biomarkers represent a potential tool for personalized DME management. While currently utilized primarily in clinical trial and research settings, future advancements in analytical platforms may facilitate the integration of these molecular insights into clinical practice. Monitoring key proteins, such as Ang-2, may offer a more granular assessment of therapeutic stability (success) than anatomical measures alone, potentially helping to identify high-risk phenotypes and guide treatment frequency. Ultimately, these biomarkers could assist in predicting long-term anatomical response and retinopathy progression, supporting more proactive and targeted management strategies in DME.

These findings reinforce the growing evidence that dual Ang-2 and VEGF-A inhibition offers a more comprehensive therapeutic strategy for DME and diabetic retinopathy compared with VEGF-A inhibition alone, linking elevated Ang-2, alongside elevated VEGF, to vascular leakage severity and inflammation.^8^^,^^9^^,^^11^^,^^18^^,^^44^, ^45^, ^46^ Our study provides evidence for the impact of faricimab on key pathologic processes, by demonstrating significant reductions in protein biomarkers of both inflammation and vascular leakage. This finding is consistent with the improved anatomical outcomes observed with faricimab in the YOSEMITE and RHINE trials, such as greater reductions in macular leakage, HRF, and epiretinal membranes compared with anti-VEGF treatment with aflibercept 2 mg.^18^ Further insight is provided by population pharmacokinetic/pharmacodynamic models, which showed that faricimab rapidly and sustainably suppresses VEGF-A and Ang-2 in the AH, whereas aflibercept did not inhibit Ang-2, confirming the distinct activity of dual inhibition.^47^

The findings of this study should be interpreted within the context of its exploratory design and certain limitations. While the sample size of 99 patients is substantial for an AH biomarker study, it remains modest for evaluating clinical efficacy endpoints compared to pivotal trials. Further, the single-arm, open-label design and the lack of a direct anti-VEGF comparator arm preclude definitive comparative conclusions regarding biomarker modulation versus monotherapy. Additionally, the fixed Q4W dosing regimen, while necessary for consistent biomarker evaluation, does not reflect the personalized T&E regimens used for faricimab in clinical practice, which may limit the direct extrapolation of these findings. From a technical perspective, the Olink proximity extension assay technology provides highly sensitive relative quantification of protein levels, which is robust for identifying changes within this study, but the lack of absolute concentrations restricts direct comparisons with data from other studies using different platforms. Finally, while our analyses adjusted for key variables, the influence of other potential confounding factors cannot be entirely excluded.^22^

## Conclusions

In treatment-naïve DME, IVT faricimab led to meaningful and consistent improvements in functional, anatomical, and morphologic outcomes across measures including BCVA, DRSS, CST, retinal fluid volume, HRF volume, and macular leakage. The safety outcomes were consistent with those reported in the YOSEMITE and RHINE phase III studies and, together, these outcomes support the favorable benefit–risk profile of faricimab. ALTIMETER uniquely integrated comprehensive AH protein profiling in all study eyes, providing a deeper understanding of the molecular changes underlying treatment response. The AH analyses revealed global normalization of protein levels by week 16, including reductions in proteins associated with macular leakage and DRSS (notably Ang-2, EPO, and PlGF), supporting our hypothesis that faricimab modulates key pathways involved in the pathology of DME. These AH proteins emerge as promising biomarkers for disease severity and treatment response, paving the way for their potential integration into future clinical trials and personalized DME management in clinical practice.
