# Supplementary material for: Aqueous Humor Biomarkers, Efficacy, and Safety in Patients with Naïve Diabetic Macular Edema Treated with Faricimab: The ALTIMETER Study
Source: Ophthalmol Sci. 2026 Feb 26;6(5):101129. doi: 10.1016/j.xops.2026.101129 (PMC13123605; doi:10.1016/j.xops.2026.101129)
Supplement: Figure S1 [file mmc1.pdf]

Figure S1

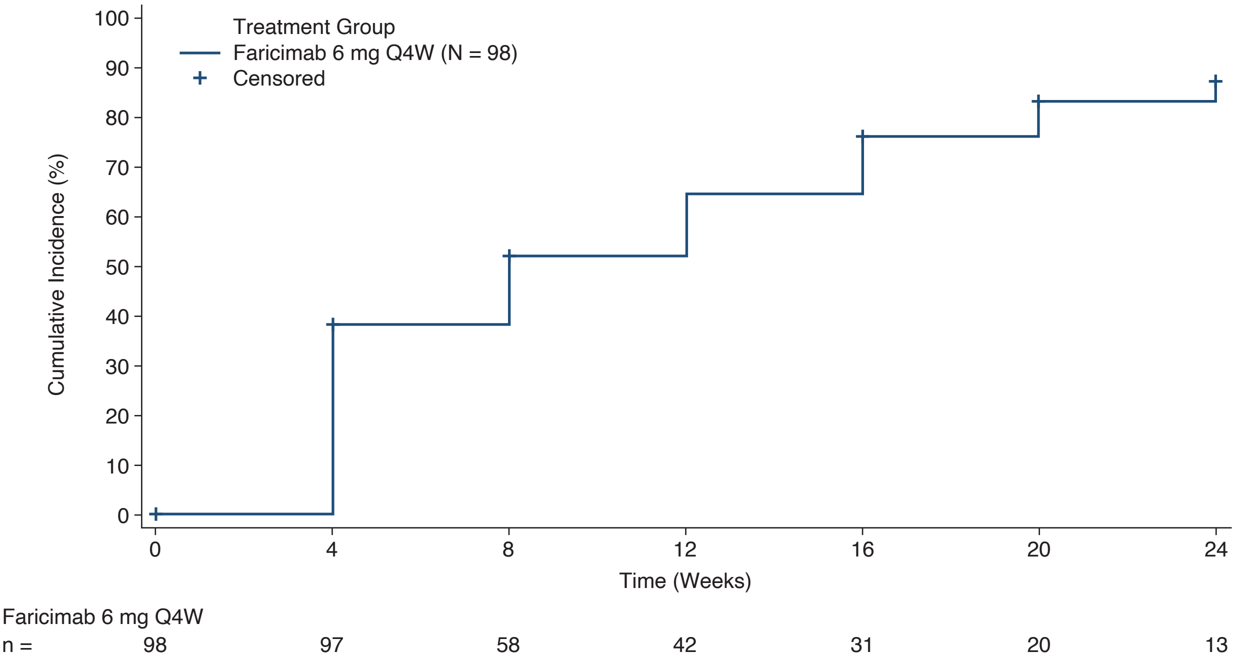

Figure shows time to first absence of DME (CST [ILM–BM] < 305 µm).
BM = Bruch's membrane; CST = central subfield thickness; DME = diabetic macular edema; ILM = internal limiting membrane; Q4W = every 4 weeks.
