# Supplementary material for: Aqueous Humor Biomarkers, Efficacy, and Safety in Patients with Naïve Diabetic Macular Edema Treated with Faricimab: The ALTIMETER Study
Source: Ophthalmol Sci. 2026 Feb 26;6(5):101129. doi: 10.1016/j.xops.2026.101129 (PMC13123605; doi:10.1016/j.xops.2026.101129)
Supplement: Figure S2 [file mmc2.pdf]

Figure S2

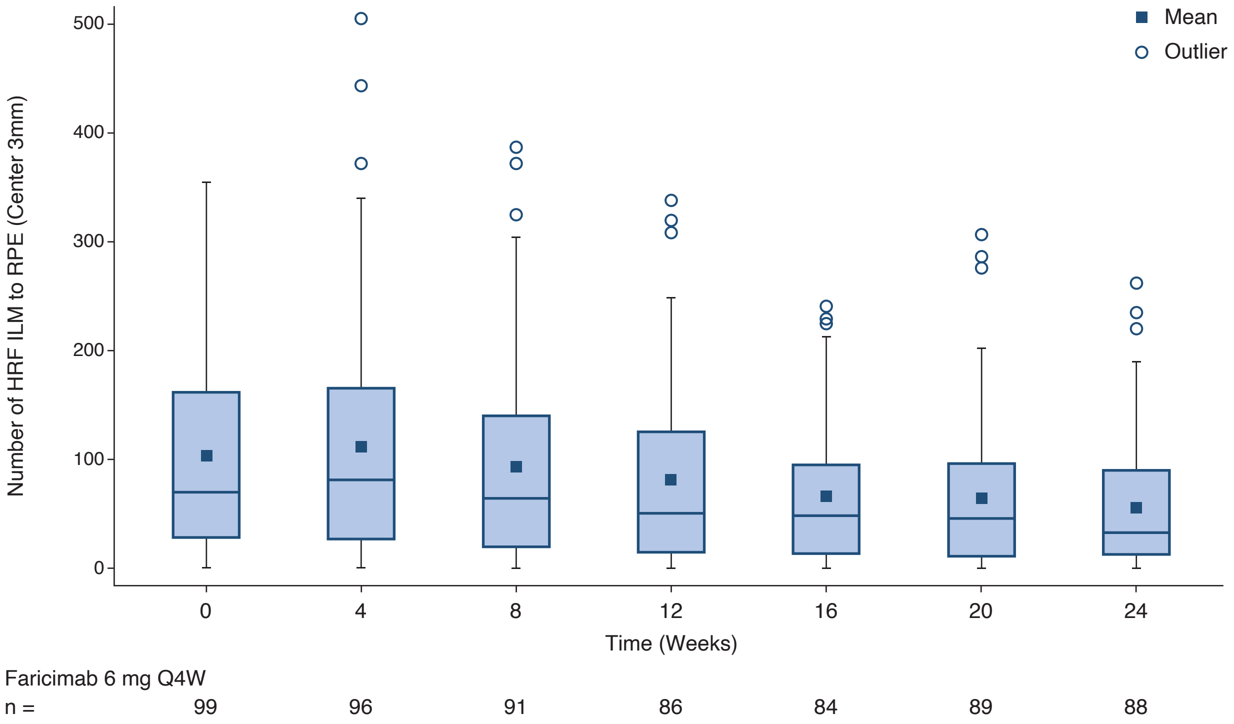

Figure shows HRF count over time, absolute values, in center 3 mm of ETDRS grid. The light blue box represents the IQR; the horizontal line within the box is at the median; and the dark blue square shows the mean value; the lines show range between minimum and maximum observations within lower (1.5[IQR] below 25th percentile) and upper (1.5[IQR] above 75th percentile) fences; the circles show outliers.

ETDRS = Early Treatment Diabetic Retinopathy Study; HRF = hyperreflective foci; ILM = internal limiting membrane; IQR = interquartile range; Q4W = every 4 weeks; RPE = retinal pigment epithelium.
