# Supplementary material for: Aqueous Humor Biomarkers, Efficacy, and Safety in Patients with Naïve Diabetic Macular Edema Treated with Faricimab: The ALTIMETER Study
Source: Ophthalmol Sci. 2026 Feb 26;6(5):101129. doi: 10.1016/j.xops.2026.101129 (PMC13123605; doi:10.1016/j.xops.2026.101129)
Supplement: Figure S3 [file mmc3.pdf]

Figure S3

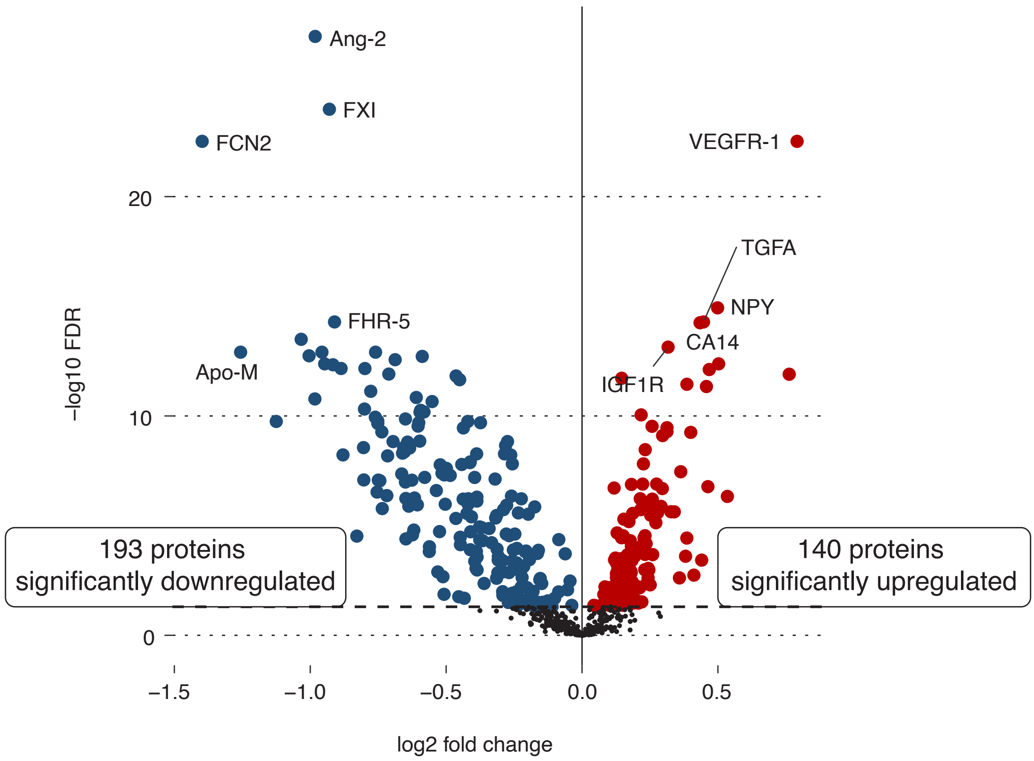

Figure shows AH protein response profile for the comparison between baseline and Week 16. Volcano plot shows the magnitude of effect (log2 fold change on the x-axis) against the statistical significance (–log10 FDR-adjusted P values on the y-axis) for the lens status-adjusted analysis. The proteins with the 10 lowest P values are labeled and the numbers of significantly up- and downregulated proteins are indicated (FDR-adjusted P value < 0.05).

AH = aqueous humor; Ang-2 = angiopoietin-2; ANGPTL4 = angiopoietin-like 4; Apo-M = apolipoprotein M; CA14 = carbonic anhydrase 14; FCN2 = ficolin 2; FDR = false discovery rate; FHR-5 = factor H-related protein 5; FXI = factor XI; IGF1R = insulin-like growth factor 1 receptor; NPY = neuropeptide Y; TGFA = transforming growth factor alpha; VEGFR-1 vascular endothelial growth factor receptor 1.
