# Supplementary material for: Aqueous Humor Biomarkers, Efficacy, and Safety in Patients with Naïve Diabetic Macular Edema Treated with Faricimab: The ALTIMETER Study
Source: Ophthalmol Sci. 2026 Feb 26;6(5):101129. doi: 10.1016/j.xops.2026.101129 (PMC13123605; doi:10.1016/j.xops.2026.101129)
Supplement: Figure S4 [file mmc4.pdf]

Figure S4A

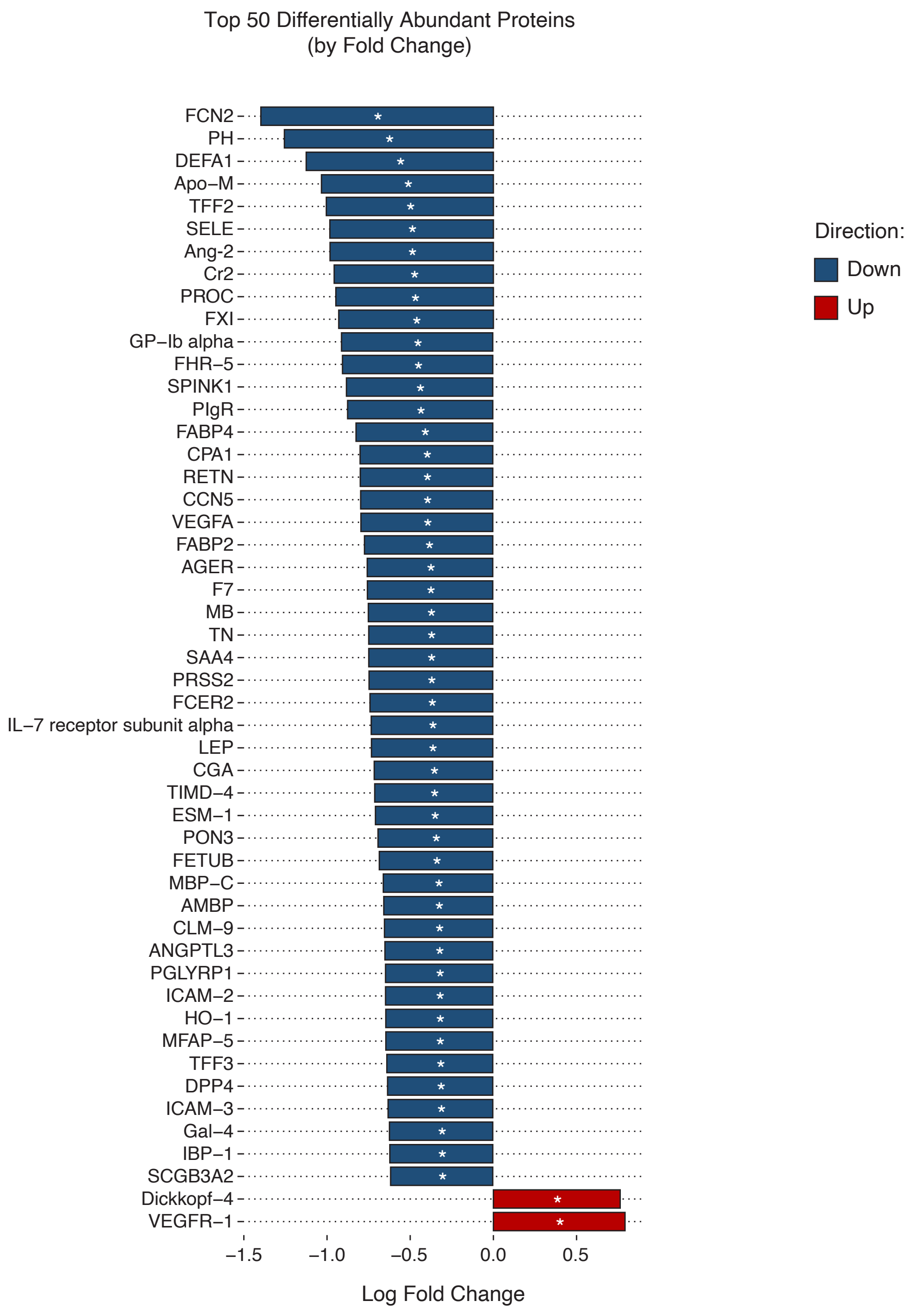

Figure S4B

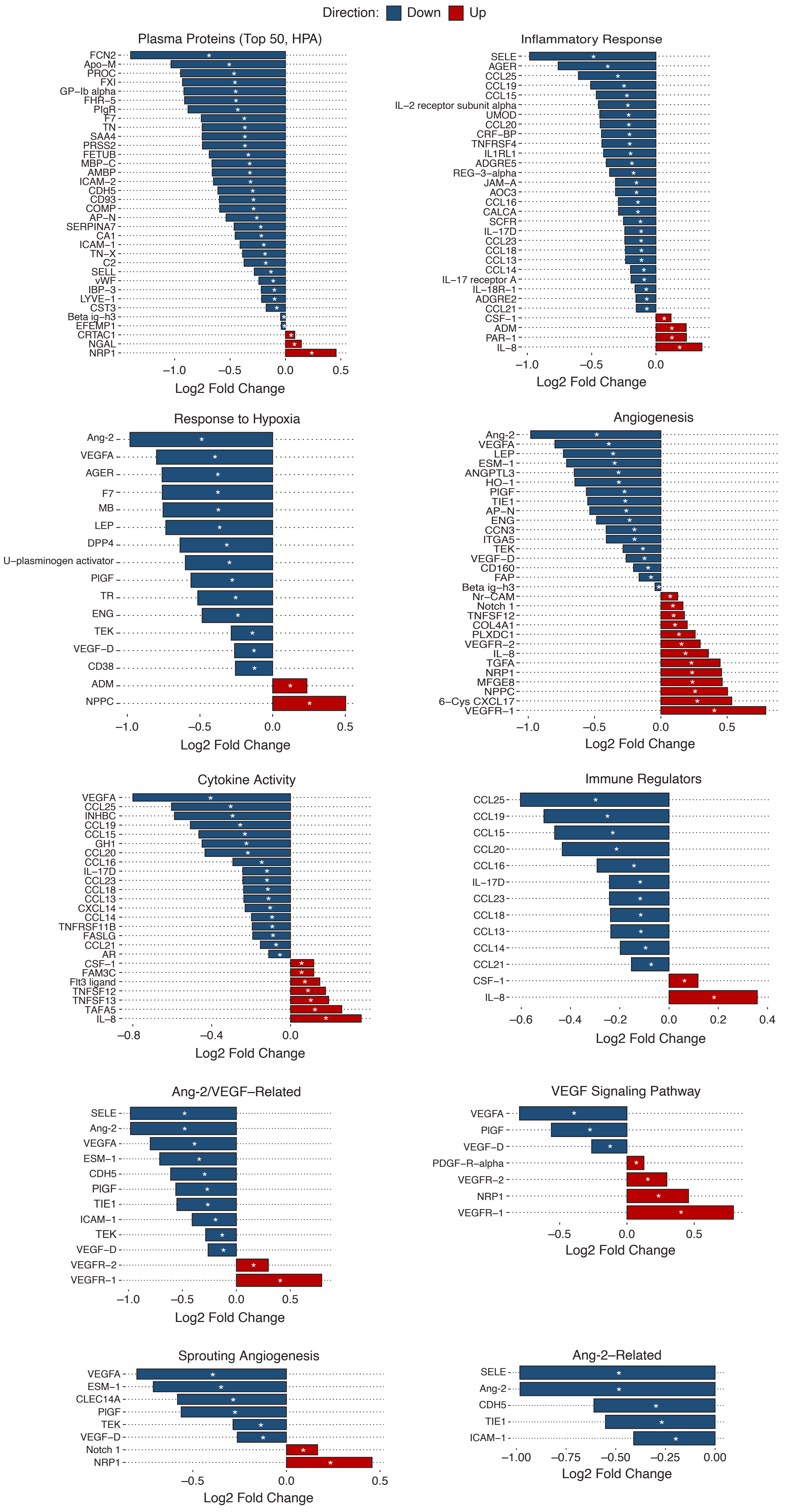

Figure shows (A) the top 50 differentially abundant proteins between baseline and Week 16, mITT population; (B) responses of the differentially abundant proteins by log2 fold change between baseline and Week 16 for the considered protein categories. Only patients with an assessment at baseline and at least 1 post-baseline value are included in this analysis. mITT population: all patients enrolled in ALTIMETER who received any amount of study treatment. Ang-2 = angiopoietin-2; HPA = Human Protein Atlas; mITT = modified intent-to-treat; VEGF = vascular endothelial growth factor.
