# Supplementary material for: Aqueous Humor Biomarkers, Efficacy, and Safety in Patients with Naïve Diabetic Macular Edema Treated with Faricimab: The ALTIMETER Study
Source: Ophthalmol Sci. 2026 Feb 26;6(5):101129. doi: 10.1016/j.xops.2026.101129 (PMC13123605; doi:10.1016/j.xops.2026.101129)
Supplement: Figure S5 [file mmc5.pdf]

Figure S5A

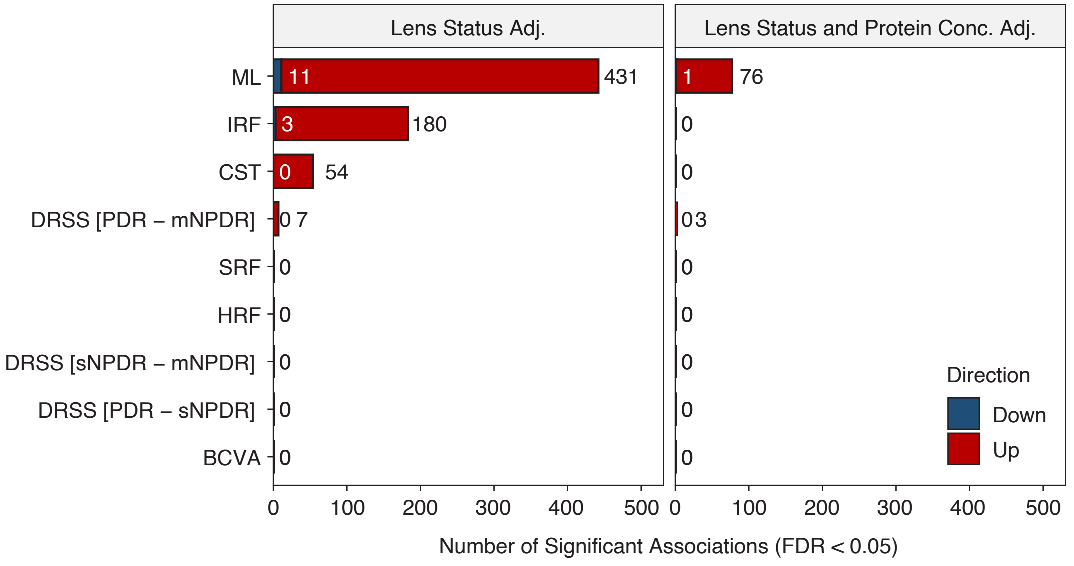

Figure S5B

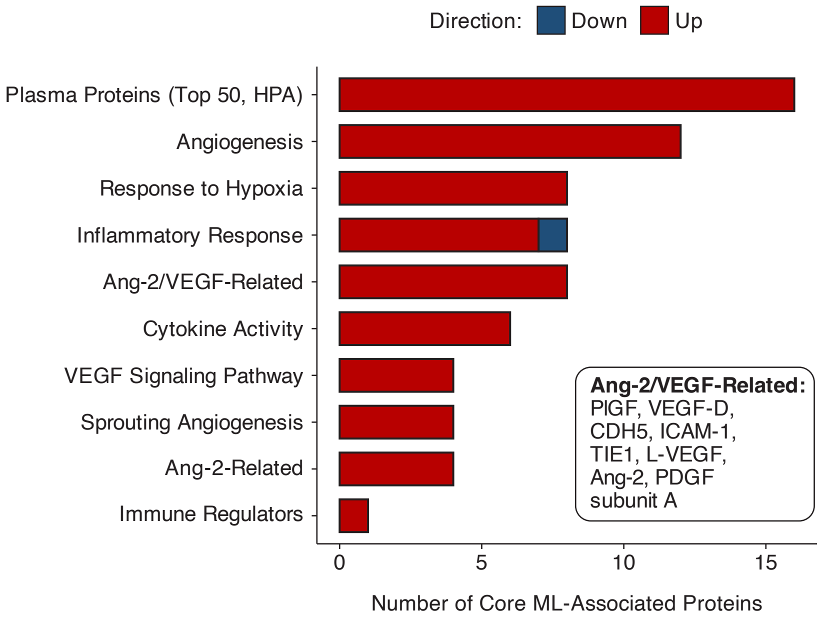

Figure shows AH protein profile and anatomical/functional parameter associations.
(A) Number of AH proteins significantly associated with each parameter at baseline (FDR < 0.05). Except for DRSS, patients with values above or below the median parameter value were compared. DRSS comparisons were performed across 3 groups (mild/moderate NPDR [mNPDR]; moderately severe/severe NPDR [sNPDR]; and PDR). The left panel shows results adjusted for lens status and the right panel shows results also adjusted for total protein concentration. The direction of the association is color-coded: up, positive association with parameter; down, negative association with parameter.
(B) Core macular-leakage-associated proteins and their associations with different parameters. These proteins correspond to those associated with macular leakage upon total protein concentration adjustment in panel A. As examples, Ang-2/VEGF-related proteins are listed.
Adj = adjusted; AH = aqueous humor; Ang-2 = angiopoietin-2; BCVA = best-corrected visual acuity; CDH5 = cadherin-5; CST = central subfield thickness; Conc. = concentration; DRSS = Diabetic Retinopathy Severity Scale; FDR = false discovery rate; HPA = Human Protein Atlas; HRF = hyperreflective foci; ICAM-1 = intracellular adhesion molecule 1; IRF = intraretinal fluid; m = moderate; ML = macular leakage; mITT = modified intent-to-treat; NPDR = non-proliferative diabetic retinopathy; PDGF = platelet-derived growth factor; PDR = proliferative diabetic retinopathy; PlGF, placental growth factor; s = severe; SRF = subretinal fluid; TIE1 = tyrosine kinase with immunoglobulin and epidermal growth factor homology domains; VEGF = vascular endothelial growth factor.
