# Supplementary material for: Aqueous Humor Biomarkers, Efficacy, and Safety in Patients with Naïve Diabetic Macular Edema Treated with Faricimab: The ALTIMETER Study
Source: Ophthalmol Sci. 2026 Feb 26;6(5):101129. doi: 10.1016/j.xops.2026.101129 (PMC13123605; doi:10.1016/j.xops.2026.101129)
Supplement: Figure S6 [file mmc6.pdf]

Figure S6

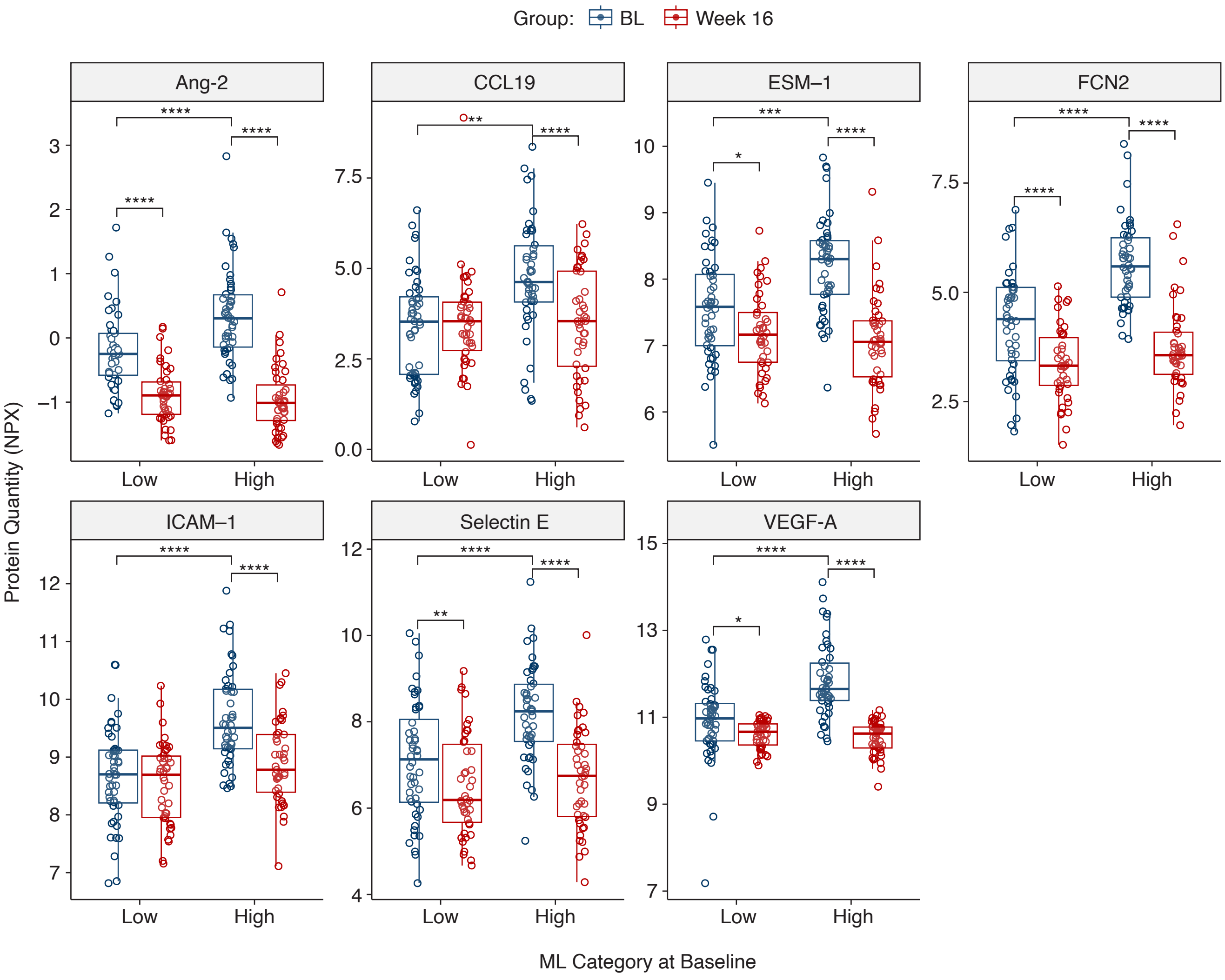

Figure shows AH protein profile patterns for patients with high vs. low ML. Box plots show unadjusted protein quantities (NPX values) for selected proteins with patients grouped by the ML category at baseline (low, ≤ median; high, > median). Individual data points and statistical significance markers are included (*FDR < 0.05, **FDR < 0.01, ***FDR < 0.001, ****FDR < 0.0001).
AH = aqueous humor; Ang-2 = angiopoietin-2; BL = baseline; CCL19 = C-C motif chemokine ligand 19; ESM-1 = endothelial cell-specific molecule 1; FCN2 = ficolin-2; FDR = false discovery rate; ICAM-1 = intercellular adhesion molecule 1; ML = macular leakage; NPX = normalized protein expression; VEGF-A = vascular endothelial growth factor-A.
