# Supplementary material for: Aqueous Humor Biomarkers, Efficacy, and Safety in Patients with Naïve Diabetic Macular Edema Treated with Faricimab: The ALTIMETER Study
Source: Ophthalmol Sci. 2026 Feb 26;6(5):101129. doi: 10.1016/j.xops.2026.101129 (PMC13123605; doi:10.1016/j.xops.2026.101129)
Supplement: Table S1 [file mmc7.pdf]

Supplementary Table S1. ALTIMETER Eligibility Criteria.

**Inclusion criteria**	
General inclusion criteria	● Signed ICF prior to study-related assessments<br>○ All patients are able and willing to provide written informed consent and to comply with the study protocol according to ICH and local regulations<br>○ Patients are willing to allow AH collection and, in the opinion of the investigator, sampling of > 90 μL of AH seems feasible and safe<br>● Ability to comply with study protocol in the investigator's judgment<br>● Age ≥ 18 years<br>● Diagnosis of diabetes mellitus (type 1 or type 2), as defined by the WHO or American Diabetes Association and:<br>○ Current regular use of insulin or other injectable drugs (e.g., dulaglutide and liraglutide) for the treatment of diabetes mellitus; and/or<br>○ Current regular use of oral anti-hyperglycemic agents for the treatment of diabetes<br>● HbA1c ≤ 10% (historic values up to 2 months before the screening visit will be permissible; otherwise, the study site may collect a sample for analysis at screening)<br>● IVT treatment-naïve in the study eye (i.e., have not received previous treatment with any anti-VEGF IVT or any corticosteroids periocular or IVT in the study eye)<br>● For patients of childbearing potential: agreement to remain abstinent (refrain from heterosexual intercourse) or use contraception as defined below:

	○ Patients must remain abstinent or use contraceptive methods with a failure rate of < 1% per year during the treatment period and for at least 3 months after the final dose of faricimab<br>○ A patient is considered to be of childbearing potential if they are post-menarchal, have not reached a postmenopausal state (≥ 12 continuous months of amenorrhea with no identified cause other than menopause), and are not permanently infertile due to surgery (i.e., removal of ovaries, fallopian tubes, and/or uterus) or another cause as determined by the investigator (e.g., Müllerian agenesis). The definition of childbearing potential may be adapted for alignment with local guidelines or regulations<br>○ Examples of contraceptive methods with a failure rate of < 1% per year include bilateral tubal ligation, male sterilization, hormonal contraceptives that inhibit ovulation, hormone-releasing intrauterine devices, and copper intrauterine devices<br>○ Contraception methods that do not result in a failure rate of < 1% per year such as male or female condom with or without spermicide; and cap, diaphragm, or sponge with spermicide are not acceptable<br>○ The reliability of sexual abstinence should be evaluated in relation to the duration of the clinical study and the preferred and usual lifestyle of the patient. Periodic abstinence (e.g., calendar, ovulation, symptothermal, or post-ovulation methods) and withdrawal are not adequate methods of contraception. If required per local guidelines or regulations, locally recognized adequate methods of contraception and information about the reliability of abstinence will be described in the local ICF
Ocular inclusion criteria	● DME defined as macular thickening by SD-OCT involving the center of the macula: CST of ≥ 325 μm with SPECTRALIS® (Heidelberg Engineering, Heidelberg, Germany) at screening This inclusion criterion is to be assessed by the CRC

	• Decreased VA is primarily attributable to DME, with BCVA letter score of 20–75 letters (both inclusive) on ETDRS-like charts at the screening visit<br>• Clear ocular media and adequate pupillary dilation to allow acquisition of good quality retinal images to confirm diagnosis
**Exclusion criteria**	
General exclusion criteria	• Currently untreated diabetes mellitus or previously untreated patients who initiated oral or injectable anti-diabetic medication within 3 months prior to Day 1<br>• Any known hypersensitivity to any of the components in the faricimab injection<br>• Any known hypersensitivity to any contrast media (e.g., fluorescein), dilating eye drops, or any of the anesthetics and antimicrobial preparations used by the patient during the study<br>• Any major illness or major surgical procedure within 1 month prior to Day 1. One re-screening for this criterion is permitted<br>• History of other diseases, other non-diabetic metabolic dysfunction, physical examination finding, historical or current clinical laboratory finding giving reasonable suspicion of a condition that contraindicates the use of the faricimab or that might affect interpretation of the results of the study or renders the patient at high-risk for treatment complications, in the opinion of the investigator<br>• Active cancer within the 12 months prior to Day 1 except for appropriately treated carcinoma in situ of the cervix, non-melanoma skin carcinoma, and prostate cancer with a Gleason score of ≤ 6 and a stable prostate-specific antigen for > 12 months<br>• Stroke or myocardial infarction within the 12 months prior to Day 1. One re-screening for this criterion is permitted

- Any febrile illness within 1 week prior to Day 1. One re-screening for this criterion is permitted
- Pregnant or breastfeeding, or intending to become pregnant during the study or within 3 months after the final dose of faricimab
  - Patients of childbearing potential must have a negative serum pregnancy test result within 28 days prior to initiation of faricimab and a negative urine pregnancy test at the baseline visit
- Uncontrolled BP; defined as systolic > 180 mmHg and/or diastolic > 100 mmHg (while patient at rest). If a patient's initial reading exceeds these values, a second reading may be taken ≥ 30 minutes later on the same day. If the patient's BP is controlled by antihypertensive medication, the patient should be taking the same medication continuously for at least 30 days prior to Day 1. One re-screening for this criterion is permitted
- Renal failure requiring renal transplant, hemodialysis, or peritoneal dialysis within 6 months prior to Day 1 or anticipated to require hemodialysis or peritoneal dialysis at any time during the study
- Any condition resulting in a compromised immune system that is likely to impact the aqueous humor inflammatory biomarkers. In case of doubt, the investigator should consult with the Medical Monitor

Prior/concomitant therapy

- Patients who are currently enrolled in or have participated in any other clinical study involving an investigational product or device, or in any other type of medical research, within 3 months or 5 half-lives (whichever is longer) prior to Day 1 and up to completion of the current study. One re-screening for this criterion is permitted
- Substance abuse occurring within 12 months prior to screening, in the investigator's judgment

	• Use of systemic immunomodulatory treatments (e.g., IL-6 inhibitors) within 6 months or 5 half-lives (whichever is longer) prior to Day 1. One re-screening for this criterion is permitted<br><br>• Use of any systemic corticosteroids (including inhaled corticosteroids from inhalers used regularly, e.g., pulmonary disease, asthma, or seasonal allergy) within 1 month prior to Day 1. One re-screening for this criterion is permitted. Note: Participants using inhaled corticosteroids occasionally (PRN); PRN use of inhaled corticosteroids is permitted if a 3-day period of abstinence between corticosteroid inhalation and study visit is maintained<br><br>• Systemic treatment for suspected or active systemic infection. Note: Ongoing use of prophylactic antibiotic therapy may be acceptable but must be in consultation with the Medical Monitor<br><br>• Any prior or concomitant systemic anti-VEGF treatment within 6 months or 5 half-lives (whichever is longer) prior to Day 1. One re-screening for this criterion is permitted<br><br>• Use of systemic medications known to be toxic to the lens, retina, or optic nerve (e.g., deferoxamine, chloroquine/hydroxychloroquine, tamoxifen, phenothiazines, or ethambutol) used during the 6-month period or 5 half-lives (whichever is longer) prior to Day 1 or likely need to be used. One re-screening for this criterion is permitted<br><br>• Received a blood transfusion within 3 months prior to the screening visit. One re-screening for this criterion is permitted<br><br>• Received any treatment that leads to immunosuppression within 6 months or 5 half-lives (whichever is longer) prior to Day 1. One re-screening for this criterion is permitted
Ocular exclusion criteria for the study eye	• High-risk PDR defined as ETDRS DRSS above 71 A (Ip et al. 2012)[1]. This exclusion criterion is to be assessed by the CRC

- Any history of or ongoing rubeosis iridis
- Any panretinal photocoagulation or macular laser photocoagulation treatment received in the study eye prior to the screening visit or expected to be received between the screening visit and Day 1
- Any history of treatment with anti-VEGF or any periocular or IVT corticosteroids in the study eye and no such treatment planned for the time between screening and Day 1
- Any treatment for dry eye disease in the last month prior to Day 1 (e.g., cyclosporine eye drops, lifitegrast eye drops). Lubricating eye drops and ointments are permitted. One re-screening for this criterion is permitted
- Any treatment with anti-inflammatory eye drops (e.g., doxycycline) within 1 month prior to Day 1. One re-screening for this criterion is permitted
- Any intraocular surgery (e.g., cataract surgery) within 3 months prior to Day 1 or any planned surgery during the study. One re-screening for this criterion is permitted
- Any glaucoma surgery/laser procedure involving the iris, trabecular meshwork, or ciliary body prior to the screening visit. Only iris surgery/laser might be allowed if they occurred more than 6 months prior to Day 1.
- History of vitreoretinal surgery/pars plana vitrectomy, corneal transplant, or radiotherapy
- Uncontrolled glaucoma (e.g., progressive loss of visual fields or defined as IOP 25 mmHg at the screening visit despite treatment with anti-glaucoma medication)
- Any active or suspected ocular or periocular infections on Day 1 (i.e., any active infectious or noninfectious conjunctivitis, keratitis, scleritis, or endophthalmitis).
- Any presence of active intraocular inflammation on Day 1 (i.e., SUN criteria 0 or NEI vitreous haze grading 0) or any history of intraocular inflammation

	• Any history of idiopathic, infectious, or noninfectious uveitis<br>• Any current or history of ocular disease other than DME that may confound assessment of the macula or affect central vision (e.g., age-related macular degeneration, retinal vein occlusion, angioid streaks, histoplasmosis, active or inactive cytomegalovirus retinitis, pathological myopia, retinal detachment, macular traction, macular hole, significant cataract, epiretinal membrane) and could either: require medical or surgical intervention during the study period to prevent or treat visual loss that might result from that condition; or preclude any visual improvement due to substantial structural damage; or preclude, in the opinion of the investigator, acquisition of good quality retinal images to confirm diagnosis.<br>• Any current ocular condition or other causes of visual impairment for which, in the opinion of the investigator, VA loss would not improve from resolution of macular edema (e.g., foveal atrophy, pigment abnormalities, dense sub-foveal hard exudates, and non-retinal condition)
Ocular exclusion criteria for the fellow eye (non-study eye)	• Patient is currently receiving treatment with brolucizumab or bevacizumab in the non-study eye and is unwilling to switch to a protocol allowed non-study eye treatment during the study<br>• Any previous treatment with Iluvien® or Retisert® (fluocinolone acetonide IVT implant) in the non-study eye<br>• If patients have been treated with corticosteroids (periocular or IVT) in the non-study eye in the past, the following washout periods prior to the screening visit would apply:<br>○ Periocular or IVT corticosteroids:<br>– Triamcinolone: 6 months;<br>– Ozurdex (dexamethasone IVT implant): 6 months<br>• Non-functioning non-study eye, defined as either:

	○ BCVA of hand motion or worse<br>○ No physical presence on non-study eye (i.e., monocular)<br>○ Legally blind in the patient's relevant jurisdiction

AH = aqueous humor; BCVA = best-corrected visual acuity; BP = blood pressure; CRC, central reading center; CST = central subfield thickness; DME = diabetic macular edema; DRSS, diabetic retinopathy severity scale; ETDRS = Early Treatment Diabetic Retinopathy Study; HbA1c = hemoglobin A1c; ICF = informed consent form; ICH = International Council for Harmonisation; IL = interleukin; IOP, intraocular pressure; IVT = intravitreal; NEI = National Eye Institute; PDR = proliferative diabetic retinopathy; PRN = pro re nata; SD-OCT = spectral-domain optical coherence tomography; SUN = standardization of uveitis nomenclature; VA = visual acuity; VEGF = vascular endothelial growth factor; WHO = World Health Organization.

1. Ip MS, Domalpally A, Hopkins JJ, et al. Long-term effects of ranibizumab on diabetic retinopathy severity and progression. *Arch Ophthalmol.* 2012;130(9):1145-1152.
