# Supplementary material for: Aqueous Humor Biomarkers, Efficacy, and Safety in Patients with Naïve Diabetic Macular Edema Treated with Faricimab: The ALTIMETER Study
Source: Ophthalmol Sci. 2026 Feb 26;6(5):101129. doi: 10.1016/j.xops.2026.101129 (PMC13123605; doi:10.1016/j.xops.2026.101129)
Supplement: Table S2 [file mmc8.pdf]

Supplementary Table S2. Proteins quantified by the Olink Target 96 panels, which were used in the AH protein analyses.

	OlinkID	UniProt	Symbol	ProteinName
Olink CARDIOMETABOLIC v.3604	OID01272	P42785	PRCP	PRCP
Olink CARDIOMETABOLIC v.3604	OID01223	P00915	CA1	CA1
Olink CARDIOMETABOLIC v.3604	OID01230	P05362	ICAM1	ICAM-1
Olink CARDIOMETABOLIC v.3604	OID01216	O00533	CHL1	CHL1
Olink CARDIOMETABOLIC v.3604	OID01291	Q15582	TGFBI	Beta ig-h3
Olink CARDIOMETABOLIC v.3604	OID01254	P17813	ENG	ENG
Olink CARDIOMETABOLIC v.3604	OID01275	P55058	PLTP	PLTP
Olink CARDIOMETABOLIC v.3604	OID01232	P05543	SERPINA7	SERPINA7
Olink CARDIOMETABOLIC v.3604	OID01255	P17936	IGFBP3	IBP-3
Olink CARDIOMETABOLIC v.3604	OID01258	P20023	CR2	Cr2
Olink CARDIOMETABOLIC v.3604	OID01229	P05154	SERPINA5	SERPINA5
Olink CARDIOMETABOLIC v.3604	OID01219	O75015	FCGR3B	FCGR3B
Olink CARDIOMETABOLIC v.3604	OID01264	P24592	IGFBP6	IBP-6
Olink CARDIOMETABOLIC v.3604	OID01245	P12830	CDH1	CDH1
Olink CARDIOMETABOLIC v.3604	OID01246	P13501	CCL5	CCL5
Olink CARDIOMETABOLIC v.3604	OID01292	Q16627	CCL14	CCL14
Olink CARDIOMETABOLIC v.3604	OID01262	P22749	GNLY	GNLY
Olink CARDIOMETABOLIC v.3604	OID01273	P46531	NOTCH1	Notch 1
Olink CARDIOMETABOLIC v.3604	OID01256	P19021	PAM	PAM
Olink CARDIOMETABOLIC v.3604	OID01228	P04070	PROC	PROC
Olink CARDIOMETABOLIC v.3604	OID01225	P01034	CST3	CST3
Olink CARDIOMETABOLIC v.3604	OID01247	P13591	NCAM1	N-CAM-1
Olink CARDIOMETABOLIC v.3604	OID01289	Q15113	PCOLCE	PCOLCE
Olink CARDIOMETABOLIC v.3604	OID01297	Q8NHL6	LILRB1	LIR-1
Olink CARDIOMETABOLIC v.3604	OID01238	P08581	MET	HGF receptor
Olink CARDIOMETABOLIC v.3604	OID01288	Q14767	LTBP2	LTBP-2
Olink CARDIOMETABOLIC v.3604	OID01253	P16871	IL7R	IL-7 receptor subunit alpha
Olink CARDIOMETABOLIC v.3604	OID01257	P19320	VCAM1	V-CAM 1
Olink CARDIOMETABOLIC v.3604	OID01249	P14151	SELL	SELL
Olink CARDIOMETABOLIC v.3604	OID01227	P03951	F11	FXI
Olink CARDIOMETABOLIC v.3604	OID01274	P49747	COMP	COMP
Olink CARDIOMETABOLIC v.3604	OID01261	P22748	CA4	CA4
Olink CARDIOMETABOLIC v.3604	OID01284	Q13332	PTPRS	R-PTP-S
Olink CARDIOMETABOLIC v.3604	OID01243	P11226	MBL2	MBP-C
Olink CARDIOMETABOLIC v.3604	OID01224	P01033	TIMP1	TIMP1
Olink CARDIOMETABOLIC v.3604	OID01306	Q9Y5C1	ANGPTL3	ANGPTL3
Olink CARDIOMETABOLIC v.3604	OID01280	Q06141	REG3A	REG-3-alpha
Olink CARDIOMETABOLIC v.3604	OID01222	P00441	SOD1	SOD1
Olink CARDIOMETABOLIC v.3604	OID01251	P15529	CD46	CD46
Olink CARDIOMETABOLIC v.3604	OID01242	P11215	ITGAM	ITGAM
Olink CARDIOMETABOLIC v.3604	OID01265	P24821	TNC	TN
Olink CARDIOMETABOLIC v.3604	OID01250	P14543	NID1	NID-1
Olink CARDIOMETABOLIC v.3604	OID01302	Q9BXR6	CFHR5	FHR-5

Olink CARDIOMETABOLIC v.3604	OID01287	Q14515	SPARCL1	SPARCL1
Olink CARDIOMETABOLIC v.3604	OID01218	O15031	PLXNB2	PLXNB2
Olink CARDIOMETABOLIC v.3604	OID01303	Q9H1U4	MEGF9	Multiple EGF-like domains protein 9
Olink CARDIOMETABOLIC v.3604	OID01226	P03950	ANG	ANG
Olink CARDIOMETABOLIC v.3604	OID01252	P15907	ST6GAL1	Alpha 2,6-ST 1
Olink CARDIOMETABOLIC v.3604	OID01266	P27487	DPP4	DPP4
Olink CARDIOMETABOLIC v.3604	OID01231	P05451	REG1A	REG1A
Olink CARDIOMETABOLIC v.3604	OID01293	Q16769	QPCT	QPCT
Olink CARDIOMETABOLIC v.3604	OID01290	Q15485	FCN2	FCN2
Olink CARDIOMETABOLIC v.3604	OID01305	Q9UGM5	FETUB	FETUB
Olink CARDIOMETABOLIC v.3604	OID01263	P23141	CES1	CES1
Olink CARDIOMETABOLIC v.3604	OID01304	Q9NQ79	CRTAC1	CRTAC1
Olink CARDIOMETABOLIC v.3604	OID01259	P20062	TCN2	TC-2
Olink CARDIOMETABOLIC v.3604	OID01236	P07478	PRSS2	PRSS2
Olink CARDIOMETABOLIC v.3604	OID01267	P32942	ICAM3	ICAM-3
Olink CARDIOMETABOLIC v.3604	OID01269	P35542	SAA4	SAA4
Olink CARDIOMETABOLIC v.3604	OID01299	Q96KN2	CNDP1	CNDP1
Olink CARDIOMETABOLIC v.3604	OID01244	P12318	FCGR2A	IgG Fc receptor II-a
Olink CARDIOMETABOLIC v.3604	OID01217	O14786	NRP1	NRP1
Olink CARDIOMETABOLIC v.3604	OID01281	Q12805	EFEMP1	EFEMP1
Olink CARDIOMETABOLIC v.3604	OID01298	Q96H15	TIMD4	TIMD-4
Olink CARDIOMETABOLIC v.3604	OID01282	Q12884	FAP	FAP
Olink CARDIOMETABOLIC v.3604	OID01270	P35590	TIE1	TIE1
Olink CARDIOMETABOLIC v.3604	OID01268	P35443	THBS4	THBS4
Olink CARDIOMETABOLIC v.3604	OID01239	P08709	F7	F7
Olink CARDIOMETABOLIC v.3604	OID01234	P07359	GP1BA	GP-Ib alpha
Olink CARDIOMETABOLIC v.3604	OID01307	Q9Y5Y7	LYVE1	LYVE-1
Olink CARDIOMETABOLIC v.3604	OID01235	P07451	CA3	CA3
Olink CARDIOMETABOLIC v.3604	OID01279	Q03167	TGFBR3	TGF-beta receptor type 3
Olink CARDIOMETABOLIC v.3604	OID01277	P59665	DEFA1	DEFA1
Olink CARDIOMETABOLIC v.3604	OID01248	P13987	CD59	CD59
Olink CARDIOMETABOLIC v.3604	OID01221	O95445	APOM	Apo-M
Olink CARDIOMETABOLIC v.3604	OID01300	Q99650	OSMR	OSMR
Olink CARDIOMETABOLIC v.3604	OID01296	Q8N423	LILRB2	LIR-2
Olink CARDIOMETABOLIC v.3604	OID01237	P07911	UMOD	UMOD
Olink CARDIOMETABOLIC v.3604	OID01276	P55774	CCL18	CCL18
Olink CARDIOMETABOLIC v.3604	OID01271	P39060	COL18A1	COL18A1
Olink CARDIOMETABOLIC v.3604	OID01278	P80188	LCN2	NGAL
Olink CARDIOMETABOLIC v.3604	OID01241	P10721	KIT	SCFR
Olink CARDIOMETABOLIC v.3604	OID01301	Q9BXJ1	C1QTNF1	C1QTNF1
Olink CARDIOMETABOLIC v.3604	OID01294	Q16853	AOC3	AOC3
Olink CARDIOMETABOLIC v.3604	OID01286	Q14393	GAS6	GAS-6
Olink CARDIOMETABOLIC v.3604	OID01240	P0DOY2	IGLC2	IGLC2
Olink CARDIOMETABOLIC v.3604	OID01283	Q13093	PLA2G7	PAF acetylhydrolase
Olink CARDIOMETABOLIC v.3604	OID01260	P22105	TNXB	TN-X
Olink CARDIOMETABOLIC v.3604	OID01285	Q13361	MFAP5	MFAP-5
Olink CARDIOMETABOLIC v.3604	OID01295	Q6EMK4	VASN	VASN
Olink CARDIOMETABOLIC v.3604	OID01220	O75023	LILRB5	LILRB5

Olink CARDIOMETABOLIC v.3604	OID01233	P06681	C2	C2
Olink CELL REGULATION v.3703	OID01382	Q9H156	SLITRK2	SLITRK2
Olink CELL REGULATION v.3703	OID01362	Q6UXK5	LRRN1	LRRN1
Olink CELL REGULATION v.3703	OID01377	Q96PQ0	SORCS2	SORCS2
Olink CELL REGULATION v.3703	OID01363	Q7Z5A7	TAFA5	TAFA5
Olink CELL REGULATION v.3703	OID01365	Q86SJ2	AMIGO2	AMIGO2
Olink CELL REGULATION v.3703	OID01333	P08237	PFKM	ATP-PFK
Olink CELL REGULATION v.3703	OID01330	P02462	COL4A1	COL4A1
Olink CELL REGULATION v.3703	OID01337	P15848	ARSB	ASB
Olink CELL REGULATION v.3703	OID01375	Q96LC7	SIGLEC10	Siglec-10
Olink CELL REGULATION v.3703	OID01341	P23515	OMG	OMG
Olink CELL REGULATION v.3703	OID01335	P0CG37	CFC1	CFC1
Olink CELL REGULATION v.3703	OID01353	Q10471	GALNT2	GALNT2
Olink CELL REGULATION v.3703	OID01311	O00451	GFRA2	GDNF receptor alpha-2
Olink CELL REGULATION v.3703	OID01316	O43521-2		
Olink CELL REGULATION v.3703	OID01358	Q16653	MOG	MOG
Olink CELL REGULATION v.3703	OID01334	P09758	TACSTD2	TACSTD2
Olink CELL REGULATION v.3703	OID01390	Q9NZN5	ARHGEF12	ARHGEF12
Olink CELL REGULATION v.3703	OID01378	Q96SM3	CPXM1	CPXM1
Olink CELL REGULATION v.3703	OID01361	Q6UXD5	SEZ6L2	SEZ6L2
Olink CELL REGULATION v.3703	OID01323	O94760	DDAH1	DDAH-1
Olink CELL REGULATION v.3703	OID01389	Q9NZ53	PODXL2	PODXL2
Olink CELL REGULATION v.3703	OID01344	P47929	LGALS7	Gal-7
Olink CELL REGULATION v.3703	OID01393	Q9UBM4	OPTC	OPTC
Olink CELL REGULATION v.3703	OID01394	Q9UK85	DKKL1	DKKL1
Olink CELL REGULATION v.3703	OID01374	Q96I82	KAZALD1	KAZALD1
Olink CELL REGULATION v.3703	OID01314	O14917	PCDH17	PCDH17
Olink CELL REGULATION v.3703	OID01320	O60243	HS6ST1	HS6ST-1
Olink CELL REGULATION v.3703	OID01321	O75054	IGSF3	IgSF3
Olink CELL REGULATION v.3703	OID01398	Q9Y662	HS3ST3B1	HS3ST3B1
Olink CELL REGULATION v.3703	OID01338	P16562	CRISP2	CRISP-2
Olink CELL REGULATION v.3703	OID01345	P52564	MAP2K6	MAP kinase kinase 6
Olink CELL REGULATION v.3703	OID01322	O75354	ENTPD6	NTPDase 6
Olink CELL REGULATION v.3703	OID01379	Q9BQT9	CLSTN3	CLSTN3
Olink CELL REGULATION v.3703	OID01385	Q9NRM6	IL17RB	IL-17 receptor B
Olink CELL REGULATION v.3703	OID01349	Q02742	GCNT1	GCNT1
Olink ONCOLOGY II v.7004	OID00655	P40222	TXLNA	TXLNA
Olink ONCOLOGY II v.7004	OID00657	P16870	CPE	CPE
Olink ONCOLOGY II v.7004	OID00658	Q9UKR3	KLK13	KLK13
Olink ONCOLOGY II v.7004	OID00660	Q13421	MSLN	MSLN
Olink ONCOLOGY II v.7004	OID00661	O75888	TNFSF13	TNFSF13
Olink ONCOLOGY II v.7004	OID00663	O95407	TNFRSF6B	TNFRSF6B
Olink ONCOLOGY II v.7004	OID00664	P18827	SDC1	SYND1
Olink ONCOLOGY II v.7004	OID00665	P37173	TGFBR2	TGFR-2
Olink ONCOLOGY II v.7004	OID00667	P09326	CD48	CD48
Olink ONCOLOGY II v.7004	OID00668	O14828	SCAMP3	Secretory carrier membrane protein 3
Olink ONCOLOGY II v.7004	OID00670	P15260	IFNGR1	IFN-gamma receptor 1
Olink ONCOLOGY II v.7004	OID00671	P06756	ITGAV	ITGAV

Olink ONCOLOGY II v.7004	OID00673	Q9UBX7	KLK11	hK11
Olink ONCOLOGY II v.7004	OID00674	P35052	GPC1	GPC1
Olink ONCOLOGY II v.7004	OID00675	P48307	TFPI2	TFPI-2
Olink ONCOLOGY II v.7004	OID00676	O60259	KLK8	hK8
Olink ONCOLOGY II v.7004	OID00677	P35968	KDR	VEGFR-2
Olink ONCOLOGY II v.7004	OID00678	O95274	LYPD3	LYPD3
Olink ONCOLOGY II v.7004	OID00680	P26447	S100A4	S100A4
Olink ONCOLOGY II v.7004	OID00681	P08069	IGF1R	IGF1R
Olink ONCOLOGY II v.7004	OID00682	P04626	ERBB2	ERBB2
Olink ONCOLOGY II v.7004	OID00683	P21860	ERBB3	ERBB3
Olink ONCOLOGY II v.7004	OID00685	P09486	SPARC	SPARC
Olink ONCOLOGY II v.7004	OID00688	P09958	FURIN	FURIN
Olink ONCOLOGY II v.7004	OID00689	O00622	CCN1	CCN1
Olink ONCOLOGY II v.7004	OID00693	Q96NY8	NECTIN4	NECTIN4
Olink ONCOLOGY II v.7004	OID00694	P48023	FASLG	FASLG
Olink ONCOLOGY II v.7004	OID00695	P29317	EPHA2	EPHA2
Olink ONCOLOGY II v.7004	OID00696	P18084	ITGB5	ITGB5
Olink ONCOLOGY II v.7004	OID00697	P09382	LGALS1	Gal-1
Olink ONCOLOGY II v.7004	OID00698	Q9BYH1	SEZ6L	SEZ6L
Olink ONCOLOGY II v.7004	OID00749	Q14956	GPNMB	GPNMB
Olink ONCOLOGY II v.7004	OID00701	Q16674	MIA	MIA
Olink ONCOLOGY II v.7004	OID00702	O60911	CTSV	CTSV
Olink ONCOLOGY II v.7004	OID00703	P26842	CD27	CD27
Olink ONCOLOGY II v.7004	OID00705	Q15303	ERBB4	ERBB4
Olink ONCOLOGY II v.7004	OID00707	P78325	ADAM8	ADAM 8
Olink ONCOLOGY II v.7004	OID00708	P21589	NT5E	5'-NT
Olink ONCOLOGY II v.7004	OID00710	O00548	DLL1	DLL1
Olink ONCOLOGY II v.7004	OID00711	P21741	MDK	MK
Olink ONCOLOGY II v.7004	OID00712	P00519	ABL1	ABL1
Olink ONCOLOGY II v.7004	OID00713	Q14512	FGFBP1	FGF-BP
Olink ONCOLOGY II v.7004	OID00714	O15455	TLR3	TLR3
Olink ONCOLOGY II v.7004	OID00716	P07949	RET	RET
Olink ONCOLOGY II v.7004	OID00717	P08670	VIM	VIM
Olink ONCOLOGY II v.7004	OID00718	Q9NS68	TNFRSF19	TNFRSF19
Olink ONCOLOGY II v.7004	OID00719	Q9UBG3	CRNN	CRNN
Olink ONCOLOGY II v.7004	OID00721	O95971	CD160	CD160
Olink ONCOLOGY II v.7004	OID00722	P43489	TNFRSF4	TNFRSF4
Olink ONCOLOGY II v.7004	OID00723	Q29983,Q29980		
Olink ONCOLOGY II v.7004	OID00724	O95388	CCN4	CCN4
Olink ONCOLOGY II v.7004	OID00725	Q6UXB2	CXCL17	6-Cys CXCL17
Olink ONCOLOGY II v.7004	OID00726	P01298	PPY	PH
Olink ONCOLOGY II v.7004	OID00727	P31949	S100A11	S100A11
Olink ONCOLOGY II v.7004	OID00729	Q9NQ30	ESM1	ESM-1
Olink ONCOLOGY II v.7004	OID00731	O75144	ICOSLG	ICOSLG
Olink ONCOLOGY II v.7004	OID00732	Q14508	WFDC2	WFDC2
Olink ONCOLOGY II v.7004	OID00733	O43927	CXCL13	CXCL13
Olink ONCOLOGY II v.7004	OID00734	Q99717	SMAD5	MAD homolog 5
Olink ONCOLOGY II v.7004	OID00735	Q8TE58	ADAMTS15	ADAM-TS 15

Olink ONCOLOGY II v.7004	OID00737	Q9BXY4	RSPO3	RSPO3
Olink ONCOLOGY II v.7004	OID00738	P41439	FOLR3	FR-gamma
Olink ONCOLOGY II v.7004	OID00742	Q9Y5W5	WIF1	WIF-1
Olink ONCOLOGY II v.7004	OID00743	P10144	GZMB	GZMB
Olink ONCOLOGY II v.7004	OID00745	P04083	ANXA1	ANXA1
Olink ONCOLOGY II v.7004	OID00746	P15328	FOLR1	FR-alpha
Olink CARDIOVASCULAR II v.5006	OID00381	P35318	ADM	ADM
Olink CARDIOVASCULAR II v.5006	OID00384	P49763	PGF	PlGF
Olink CARDIOVASCULAR II v.5006	OID00386	Q9BWV1	BOC	Protein BOC
Olink CARDIOVASCULAR II v.5006	OID00389	P18510	IL1RN	IL-1RN
Olink CARDIOVASCULAR II v.5006	OID00391	O00220	TNFRSF10A	TNFRSF10A
Olink CARDIOVASCULAR II v.5006	OID00393	P35475	IDUA	IDUA
Olink CARDIOVASCULAR II v.5006	OID00394	Q9Y6Q6	TNFRSF11A	TNFRSF11A
Olink CARDIOVASCULAR II v.5006	OID00395	P25116	F2R	PAR-1
Olink CARDIOVASCULAR II v.5006	OID00396	O14763	TNFRSF10B	TNFRSF10B
Olink CARDIOVASCULAR II v.5006	OID00397	Q9BQR3	PRSS27	PRSS27
Olink CARDIOVASCULAR II v.5006	OID00398	Q02763	TEK	TEK
Olink CARDIOVASCULAR II v.5006	OID00399	P13726	F3	TF
Olink CARDIOVASCULAR II v.5006	OID00401	P01127	PDGFB	PDGF subunit B
Olink CARDIOVASCULAR II v.5006	OID00402	Q8NEV9,Q14213		
Olink CARDIOVASCULAR II v.5006	OID00403	Q8TAD2	IL17D	IL-17D
Olink CARDIOVASCULAR II v.5006	OID00405	P78380	OLR1	Ox-LDL receptor 1
Olink CARDIOVASCULAR II v.5006	OID00406	O00182	LGALS9	Gal-9
Olink CARDIOVASCULAR II v.5006	OID00407	P27352	CBLIF	CBLIF
Olink CARDIOVASCULAR II v.5006	OID00411	P01833	PIGR	PIgR
Olink CARDIOVASCULAR II v.5006	OID00412	Q15109	AGER	AGER
Olink CARDIOVASCULAR II v.5006	OID00413	P04179	SOD2	SOD2
Olink CARDIOVASCULAR II v.5006	OID00414	Q99895	CTRC	CTRC
Olink CARDIOVASCULAR II v.5006	OID00416	Q9BUD6	SPON2	SPON2
Olink CARDIOVASCULAR II v.5006	OID00417	P01241	GH1	GH1
Olink CARDIOVASCULAR II v.5006	OID00418	P19883	FST	FS
Olink CARDIOVASCULAR II v.5006	OID00419	Q04760	GLO1	GLO1
Olink CARDIOVASCULAR II v.5006	OID00421	Q13219	PAPPA	PAPPA
Olink CARDIOVASCULAR II v.5006	OID00423	P00797	REN	REN
Olink CARDIOVASCULAR II v.5006	OID00424	Q16698	DECR1	DECR1
Olink CARDIOVASCULAR II v.5006	OID00427	P35442	THBS2	THBS2
Olink CARDIOVASCULAR II v.5006	OID00428	P07204	THBD	TM

Olink CARDIOVASCULAR II v.5006	OID00430	P02760	AMBP	AMBP
Olink CARDIOVASCULAR II v.5006	OID00431	P51888	PRELP	PRELP
Olink CARDIOVASCULAR II v.5006	OID00432	P09601	HMOX1	HO-1
Olink CARDIOVASCULAR II v.5006	OID00435	Q99523	SORT1	SORT1
Olink CARDIOVASCULAR II v.5006	OID00439	Q92583	CCL17	CCL17
Olink CARDIOVASCULAR II v.5006	OID00441	P09237	MMP7	MMP7
Olink CARDIOVASCULAR II v.5006	OID00444	P07585	DCN	DCN
Olink CARDIOVASCULAR II v.5006	OID00445	O94907	DKK1	Dickkopf-1
Olink CARDIOVASCULAR II v.5006	OID00446	P06858	LPL	LPL
Olink CARDIOVASCULAR II v.5006	OID00447	Q16651	PRSS8	PRSS8
Olink CARDIOVASCULAR II v.5006	OID00448	O00253	AGRP	AGRP
Olink CARDIOVASCULAR II v.5006	OID00449	Q99075	HBEGF	HBEGF
Olink CARDIOVASCULAR II v.5006	OID00451	P12104	FABP2	FABP2
Olink CARDIOVASCULAR II v.5006	OID00453	Q9UEW3	MARCO	MARCO
Olink CARDIOVASCULAR II v.5006	OID00456	P39900	MMP12	MME
Olink CARDIOVASCULAR II v.5006	OID00459	P07711	CTSL	CTSL
Olink CARDIOVASCULAR II v.5006	OID00460	Q8IYS5	OSCAR	Osteoclast-associated receptor
Olink CARDIOVASCULAR II v.5006	OID00461	O14836	TNFRSF13B	TNFRSF13B
Olink CARDIOVASCULAR II v.5006	OID00462	P21980	TGM2	TGM2
Olink CARDIOVASCULAR II v.5006	OID00463	P41159	LEP	LEP
Olink CARDIOVASCULAR II v.5006	OID00465	P04792	HSPB1	HspB1
Olink CARDIOVASCULAR II v.5006	OID00466	P01730	CD4	CD4
Olink CARDIOVASCULAR II v.5006	OID00468	O43915	VEGFD	VEGF-D
Olink CARDIOVASCULAR III v.6114	OID00563	Q92956	TNFRSF14	TNFRSF14
Olink CARDIOVASCULAR III v.6114	OID00564	P01130	LDLR	LDL receptor
Olink CARDIOVASCULAR III v.6114	OID00565	P05107	ITGB2	ITGB2
Olink CARDIOVASCULAR III v.6114	OID00566	Q96F46	IL17RA	IL-17 receptor A
Olink CARDIOVASCULAR III v.6114	OID00567	P20333	TNFRSF1B	TNFRSF1B
Olink CARDIOVASCULAR III v.6114	OID00568	P14780	MMP9	MMP-9
Olink CARDIOVASCULAR III v.6114	OID00569	P54760	EPHB4	EPHB4
Olink CARDIOVASCULAR III v.6114	OID00570	P01589	IL2RA	IL-2 receptor subunit alpha
Olink CARDIOVASCULAR III v.6114	OID00572	Q13740	ALCAM	ALCAM
Olink CARDIOVASCULAR III v.6114	OID00573	Q07654	TFF3	TFF3
Olink CARDIOVASCULAR III v.6114	OID00574	P16109	SELP	SELP
Olink CARDIOVASCULAR III v.6114	OID00575	P04080	CSTB	CSTB

Olink CARDIOVASCULAR III v.6114	OID00577	Q86VB7	CD163	CD163
Olink CARDIOVASCULAR III v.6114	OID00579	P28799	GRN	PGRN
Olink CARDIOVASCULAR III v.6114	OID00131	NT-proBNP		
Olink CARDIOVASCULAR III v.6114	OID00581	Q13867	BLMH	BH
Olink CARDIOVASCULAR III v.6114	OID00582	P98160	HSPG2	HSPG
Olink CARDIOVASCULAR III v.6114	OID00583	P36941	LTBR	LTBR
Olink CARDIOVASCULAR III v.6114	OID00584	Q9UM47	NOTCH3	Notch 3
Olink CARDIOVASCULAR III v.6114	OID00585	Q99727	TIMP4	TIMP4
Olink CARDIOVASCULAR III v.6114	OID00586	Q12860	CNTN1	CNTN1
Olink CARDIOVASCULAR III v.6114	OID00587	P33151	CDH5	CDH5
Olink CARDIOVASCULAR III v.6114	OID00588	Q5T2D2	TREML2	TLT-2
Olink CARDIOVASCULAR III v.6114	OID00589	P15090	FABP4	FABP4
Olink CARDIOVASCULAR III v.6114	OID00590	P10646	TFPI	TFPI
Olink CARDIOVASCULAR III v.6114	OID00591	P05121	SERPINE1	PAI
Olink CARDIOVASCULAR III v.6114	OID00592	O00175	CCL24	CCL24
Olink CARDIOVASCULAR III v.6114	OID00593	P02786	TFRC	TR
Olink CARDIOVASCULAR III v.6114	OID00594	O14798	TNFRSF10C	TNFRSF10C
Olink CARDIOVASCULAR III v.6114	OID00595	Q99988	GDF15	GDF-15
Olink CARDIOVASCULAR III v.6114	OID00596	P16581	SELE	SELE
Olink CARDIOVASCULAR III v.6114	OID00597	P20160	AZU1	AZU1
Olink CARDIOVASCULAR III v.6114	OID00598	P80370	DLK1	DLK-1
Olink CARDIOVASCULAR III v.6114	OID00599	Q9HCB6	SPON1	SPON1
Olink CARDIOVASCULAR III v.6114	OID00600	P05164	MPO	MPO
Olink CARDIOVASCULAR III v.6114	OID00601	Q9H2A7	CXCL16	CXCL16
Olink CARDIOVASCULAR III v.6114	OID00602	P08887	IL6R	IL-6 receptor subunit alpha
Olink CARDIOVASCULAR III v.6114	OID00603	Q9HD89	RETN	RETN
Olink CARDIOVASCULAR III v.6114	OID00604	P08833	IGFBP1	IBP-1
Olink CARDIOVASCULAR III v.6114	OID00605	Q13231	CHIT1	CHIT1
Olink CARDIOVASCULAR III v.6114	OID00606	P13686	ACP5	TR-AP
Olink CARDIOVASCULAR III v.6114	OID05026	Q9HCN6	GP6	GPVI
Olink CARDIOVASCULAR III v.6114	OID00608	P35247	SFTPD	PSP-D
Olink CARDIOVASCULAR III v.6114	OID00609	P19957	PI3	PI3
Olink CARDIOVASCULAR III v.6114	OID00610	P16422	EPCAM	Ep-CAM
Olink CARDIOVASCULAR III v.6114	OID00611	P15144	ANPEP	AP-N
Olink CARDIOVASCULAR III v.6114	OID00612	P30530	AXL	AXL

Olink CARDIOVASCULAR III v.6114	OID00613	P14778	IL1R1	IL-1R-1
Olink CARDIOVASCULAR III v.6114	OID00614	P08253	MMP2	MMP2
Olink CARDIOVASCULAR III v.6114	OID00615	P25445	FAS	FAS
Olink CARDIOVASCULAR III v.6114	OID00616	P02144	MB	MB
Olink CARDIOVASCULAR III v.6114	OID00617	Q9Y275	TNFSF13B	TNFSF13B
Olink CARDIOVASCULAR III v.6114	OID00618	P24158	PRTN3	PRTN3
Olink CARDIOVASCULAR III v.6114	OID00619	Q8NBP7	PCSK9	PCSK9
Olink CARDIOVASCULAR III v.6114	OID00620	Q03405	PLAUR	U-PAR
Olink CARDIOVASCULAR III v.6114	OID00621	P10451	SPP1	SPP1
Olink CARDIOVASCULAR III v.6114	OID00622	P07339	CTSD	CTSD
Olink CARDIOVASCULAR III v.6114	OID00623	O75594	PGLYRP1	PGLYRP1
Olink CARDIOVASCULAR III v.6114	OID00624	P15085	CPA1	CPA1
Olink CARDIOVASCULAR III v.6114	OID00625	Q9Y624	F11R	JAM-A
Olink CARDIOVASCULAR III v.6114	OID00626	P56470	LGALS4	Gal-4
Olink CARDIOVASCULAR III v.6114	OID00627	P27930	IL1R2	IL-1R-2
Olink CARDIOVASCULAR III v.6114	OID00628	P78324	SIRPA	SHP substrate 1
Olink CARDIOVASCULAR III v.6114	OID00629	Q16663	CCL15	CCL15
Olink CARDIOVASCULAR III v.6114	OID00630	P42574	CASP3	CASP-3
Olink CARDIOVASCULAR III v.6114	OID00632	P15086	CPB1	CPB1
Olink CARDIOVASCULAR III v.6114	OID00633	P36222	CHI3L1	CHI3L1
Olink CARDIOVASCULAR III v.6114	OID00634	Q01638	IL1RL1	IL1RL1
Olink CARDIOVASCULAR III v.6114	OID00635	P00750	PLAT	t-PA
Olink CARDIOVASCULAR III v.6114	OID00636	Q96PL1	SCGB3A2	SCGB3A2
Olink CARDIOVASCULAR III v.6114	OID00637	P00533	EGFR	EGFR
Olink CARDIOVASCULAR III v.6114	OID00638	Q16270	IGFBP7	IBP-7
Olink CARDIOVASCULAR III v.6114	OID00639	Q9NPY3	CD93	CD93
Olink CARDIOVASCULAR III v.6114	OID00641	P02452	COL1A1	COL1A1
Olink CARDIOVASCULAR III v.6114	OID00642	Q15166	PON3	PON3
Olink CARDIOVASCULAR III v.6114	OID00643	Q9UBR2	CTSZ	CTSZ
Olink CARDIOVASCULAR III v.6114	OID00644	P08254	MMP3	SL-1
Olink CARDIOVASCULAR III v.6114	OID00645	Q99969	RARRES2	RARRES2
Olink CARDIOVASCULAR III v.6114	OID00646	P13598	ICAM2	ICAM-2
Olink CARDIOVASCULAR III v.6114	OID00647	Q92876	KLK6	KLK6
Olink CARDIOVASCULAR III v.6114	OID00648	P04085	PDGFA	PDGF subunit A
Olink CARDIOVASCULAR III v.6114	OID00649	P19438	TNFRSF1A	TNFRSF1A

Olink CARDIOVASCULAR III v.6114	OID00650	P18065	IGFBP2	IBP-2
Olink CARDIOVASCULAR III v.6114	OID00651	P04275	VWF	vWF
Olink CARDIOVASCULAR III v.6114	OID00132	Q9NQ76	MEPE	MEPE
Olink CARDIOVASCULAR III v.6114	OID00654	O15467	CCL16	CCL16
Olink DEVELOPMENT v.3512	OID01400	Q9UBX1	CTSF	CATSF
Olink DEVELOPMENT v.3512	OID01401	O00339	MATN2	MATN2
Olink DEVELOPMENT v.3512	OID01402	O43464	HTRA2	HTRA2
Olink DEVELOPMENT v.3512	OID01403	Q08431	MFGE8	MFGE8
Olink DEVELOPMENT v.3512	OID01404	Q9UKK9	NUDT5	NUDT5
Olink DEVELOPMENT v.3512	OID01405	P16112	ACAN	ACAN
Olink DEVELOPMENT v.3512	OID01406	P05556	ITGB1	ITGB1
Olink DEVELOPMENT v.3512	OID01407	Q9UBP4	DKK3	Dickkopf-3
Olink DEVELOPMENT v.3512	OID01408	O43291	SPINT2	SPINT2
Olink DEVELOPMENT v.3512	OID01409	P05067	APP	APP
Olink DEVELOPMENT v.3512	OID01410	Q8TDQ0	HAVCR2	HAVcr-2
Olink DEVELOPMENT v.3512	OID01411	Q02487	DSC2	DSC2
Olink DEVELOPMENT v.3512	OID01412	P63313	TMSB10	TMSB10
Olink DEVELOPMENT v.3512	OID01413	Q9BY76	ANGPTL4	ANGPTL4
Olink DEVELOPMENT v.3512	OID01414	P55103	INHBC	INHBC
Olink DEVELOPMENT v.3512	OID01415	P00995	SPINK1	SPINK1
Olink DEVELOPMENT v.3512	OID01416	Q9Y240	CLEC11A	CLEC11A
Olink DEVELOPMENT v.3512	OID01417	P09619	PDGFRB	PDGF-R-beta
Olink DEVELOPMENT v.3512	OID01418	O43405	COCH	COCH
Olink DEVELOPMENT v.3512	OID01419	Q96RD9	FCRL5	FcR-like protein 5
Olink DEVELOPMENT v.3512	OID01420	Q9UKJ1	PILRA	PILRA
Olink DEVELOPMENT v.3512	OID01421	P15291	B4GALT1	Beta-1,4-GalTase 1
Olink DEVELOPMENT v.3512	OID01422	Q6UXG3	CD300LG	CLM-9
Olink DEVELOPMENT v.3512	OID01423	Q99497	PARK7	PARK7
Olink DEVELOPMENT v.3512	OID01424	P30043	BLVRB	FR
Olink DEVELOPMENT v.3512	OID01426	P04233	CD74	CD74
Olink DEVELOPMENT v.3512	OID01427	O00585	CCL21	CCL21
Olink DEVELOPMENT v.3512	OID01428	P10586	PTPRF	PTPRF
Olink DEVELOPMENT v.3512	OID01429	P50895	BCAM	BCAM
Olink DEVELOPMENT v.3512	OID01430	O00241	SIRPB1	SIRP-beta-1
Olink DEVELOPMENT v.3512	OID01431	P11717	IGF2R	CI Man-6-P receptor
Olink DEVELOPMENT v.3512	OID01432	P07237	P4HB	PDI
Olink DEVELOPMENT v.3512	OID01433	P04066	FUCA1	FUCA1
Olink DEVELOPMENT v.3512	OID01434	Q96AP7	ESAM	ESAM
Olink DEVELOPMENT v.3512	OID05025	Q13822	ENPP2	ENPP2
Olink DEVELOPMENT v.3512	OID01437	Q15828	CST6	CST6
Olink DEVELOPMENT v.3512	OID01438	Q99972	MYOC	MYOC
Olink DEVELOPMENT v.3512	OID01439	O95721	SNAP29	SNAP-29
Olink DEVELOPMENT v.3512	OID01440	Q8TEU8	WFIKKN2	WFIKKN2
Olink DEVELOPMENT v.3512	OID01441	Q4KMG0	CDON	CDON
Olink DEVELOPMENT v.3512	OID01442	Q8N6Q3	CD177	CD177
Olink DEVELOPMENT v.3512	OID01443	Q14112	NID2	NID-2
Olink DEVELOPMENT v.3512	OID01444	Q14118	DAG1	DAG1

Olink DEVELOPMENT v.3512	OID01445	P48960	ADGRE5	ADGRE5
Olink DEVELOPMENT v.3512	OID01447	Q6YHK3	CD109	CD109
Olink DEVELOPMENT v.3512	OID01448	Q9Y279	VSIG4	VSIG4
Olink DEVELOPMENT v.3512	OID01449	Q9NZV1	CRIM1	CRIM-1
Olink DEVELOPMENT v.3512	OID01450	P01215	CGA	CGA
Olink DEVELOPMENT v.3512	OID01451	Q86T13	CLEC14A	CLEC14A
Olink DEVELOPMENT v.3512	OID01452	P23280	CA6	CA6
Olink DEVELOPMENT v.3512	OID01453	P14174	MIF	MIF
Olink DEVELOPMENT v.3512	OID01454	Q6UXH1	CRELD2	CRELD2
Olink DEVELOPMENT v.3512	OID01455	P08648	ITGA5	ITGA5
Olink DEVELOPMENT v.3512	OID01456	Q5VY43	PEAR1	hPEAR1
Olink DEVELOPMENT v.3512	OID01457	Q14696	MESD	MESD
Olink DEVELOPMENT v.3512	OID01458	Q8TCZ2	CD99L2	CD99L2
Olink DEVELOPMENT v.3512	OID01459	Q14162	SCARF1	SCARF1
Olink DEVELOPMENT v.3512	OID01460	Q99538	LGMN	LGMN
Olink DEVELOPMENT v.3512	OID01461	O75326	SEMA7A	SEMA7A
Olink DEVELOPMENT v.3512	OID01462	Q5KU26	COLEC12	COLEC12
Olink DEVELOPMENT v.3512	OID01463	P08236	GUSB	GUSB
Olink DEVELOPMENT v.3512	OID01492	Q11128,P21217		
Olink DEVELOPMENT v.3512	OID01465	P31948	STIP1	STI1
Olink DEVELOPMENT v.3512	OID01466	O43505	B4GAT1	B4GAT1
Olink DEVELOPMENT v.3512	OID01467	Q07108	CD69	CD69
Olink DEVELOPMENT v.3512	OID01468	P24387	CRHBP	CRF-BP
Olink DEVELOPMENT v.3512	OID01469	P08118	MSMB	MSMB
Olink DEVELOPMENT v.3512	OID01470	O43278	SPINT1	SPINT1
Olink DEVELOPMENT v.3512	OID01471	O95633	FSTL3	FSTL3
Olink DEVELOPMENT v.3512	OID01472	P78552	IL13RA1	IL-13 receptor subunit alpha-1
Olink DEVELOPMENT v.3512	OID01473	P19256	CD58	Ag3
Olink DEVELOPMENT v.3512	OID05579	O76076	CCN5	CCN5
Olink DEVELOPMENT v.3512	OID01475	P48745	CCN3	CCN3
Olink DEVELOPMENT v.3512	OID01476	Q8IWV2	CNTN4	CNTN4
Olink DEVELOPMENT v.3512	OID01477	P00918	CA2	CA2
Olink DEVELOPMENT v.3512	OID01478	P55808	XG	XG
Olink DEVELOPMENT v.3512	OID01479	P15289	ARSA	ASA
Olink DEVELOPMENT v.3512	OID01480	P23284	PPIB	PPIase B
Olink DEVELOPMENT v.3512	OID01481	Q9NQ38	SPINK5	SPINK5
Olink DEVELOPMENT v.3512	OID01482	Q99983	OMD	OMD
Olink DEVELOPMENT v.3512	OID01483	P30086	PEBP1	PEBP-1
Olink DEVELOPMENT v.3512	OID01484	Q6UXH9	PAMR1	PAMR1
Olink DEVELOPMENT v.3512	OID01485	Q9Y6N7	ROBO1	ROBO1
Olink DEVELOPMENT v.3512	OID01486	P06734	FCER2	FCER2
Olink DEVELOPMENT v.3512	OID01487	Q16363	LAMA4	LAMA4
Olink DEVELOPMENT v.3512	OID01488	Q6GTX8	LAIR1	LAIR-1
Olink DEVELOPMENT v.3512	OID01489	Q969Z4	RELT	RELT
Olink DEVELOPMENT v.3512	OID01490	O14773	TPP1	TPP-1
Olink DEVELOPMENT v.3512	OID01491	Q9NNX6	CD209	CD209
Olink INFLAMMATION v.3022	OID00471	P10145	CXCL8	IL-8
Olink INFLAMMATION v.3022	OID00472	P15692	VEGFA	VEGFA

Olink INFLAMMATION v.3022	OID05124	P01732	CD8A	CD8A
Olink INFLAMMATION v.3022	OID00476	Q9H5V8	CDCP1	CDCP1
Olink INFLAMMATION v.3022	OID00478	P13232	IL7	IL-7
Olink INFLAMMATION v.3022	OID00479	O00300	TNFRSF11B	TNFRSF11B
Olink INFLAMMATION v.3022	OID00480	P01137	TGFB1	TGFB1
Olink INFLAMMATION v.3022	OID00481	P00749	PLAU	U-plasminogen activator
Olink INFLAMMATION v.3022	OID00482	P05231	IL6	IL-6
Olink INFLAMMATION v.3022	OID00484	P13500	CCL2	CCL2
Olink INFLAMMATION v.3022	OID00486	O14625	CXCL11	CXCL11
Olink INFLAMMATION v.3022	OID00488	P50591	TNFSF10	TNFSF10
Olink INFLAMMATION v.3022	OID00490	Q07325	CXCL9	CXCL9
Olink INFLAMMATION v.3022	OID00491	P28325	CST5	CST5
Olink INFLAMMATION v.3022	OID00494	P13725	OSM	OSM
Olink INFLAMMATION v.3022	OID00496	P09341	CXCL1	CXCL1
Olink INFLAMMATION v.3022	OID00498	P13236	CCL4	CCL4
Olink INFLAMMATION v.3022	OID00500	P21583	KITLG	KITLG
Olink INFLAMMATION v.3022	OID00501	Q14116	IL18	IL-18
Olink INFLAMMATION v.3022	OID00503	P01135	TGFA	TGFA
Olink INFLAMMATION v.3022	OID00504	Q99616	CCL13	CCL13
Olink INFLAMMATION v.3022	OID00505	P51671	CCL11	CCL11
Olink INFLAMMATION v.3022	OID00510	P03956	MMP1	MMP1
Olink INFLAMMATION v.3022	OID00512	Q9NSA1	FGF21	FGF-21
Olink INFLAMMATION v.3022	OID00513	Q99731	CCL19	CCL19
Olink INFLAMMATION v.3022	OID00515	Q08334	IL10RB	IL-10 receptor subunit beta
Olink INFLAMMATION v.3022	OID00517	Q13478	IL18R1	IL-18R-1
Olink INFLAMMATION v.3022	OID00518	Q9NZQ7	CD274	PD-L1
Olink INFLAMMATION v.3022	OID00520	P42830	CXCL5	CXCL5
Olink INFLAMMATION v.3022	OID00522	P14210	HGF	HGF
Olink INFLAMMATION v.3022	OID00523	P29460	IL12B	IL-12B
Olink INFLAMMATION v.3022	OID00527	P09238	MMP10	SL-2
Olink INFLAMMATION v.3022	OID00530	P55773	CCL23	CCL23
Olink INFLAMMATION v.3022	OID00531	P06127	CD5	CD5
Olink INFLAMMATION v.3022	OID00532	P10147	CCL3	CCL3
Olink INFLAMMATION v.3022	OID00533	P49771	FLT3LG	Flt3 ligand
Olink INFLAMMATION v.3022	OID00534	P80162	CXCL6	CXCL6
Olink INFLAMMATION v.3022	OID00535	P02778	CXCL10	CXCL10
Olink INFLAMMATION v.3022	OID00536	Q13541	EIF4EBP1	4E-BP1
Olink INFLAMMATION v.3022	OID00539	Q9NRJ3	CCL28	CCL28
Olink INFLAMMATION v.3022	OID01213	Q8NFT8	DNER	DNER
Olink INFLAMMATION v.3022	OID00542	P25942	CD40	CD40
Olink INFLAMMATION v.3022	OID00545	O95750	FGF19	FGF-19
Olink INFLAMMATION v.3022	OID00547	P15018	LIF	LIF
Olink INFLAMMATION v.3022	OID00549	P80075	CCL8	CCL8
Olink INFLAMMATION v.3022	OID00550	Q14790	CASP8	CASP-8
Olink INFLAMMATION v.3022	OID00551	O15444	CCL25	CCL25
Olink INFLAMMATION v.3022	OID00552	P78423	CX3CL1	CX3CL1
Olink INFLAMMATION v.3022	OID00553	Q07011	TNFRSF9	TNFRSF9
Olink INFLAMMATION v.3022	OID00555	O43508	TNFSF12	TNFSF12

Olink INFLAMMATION v.3022	OID00556	P78556	CCL20	CCL20
Olink INFLAMMATION v.3022	OID00560	P00813	ADA	ADA
Olink INFLAMMATION v.3022	OID00562	P09603	CSF1	CSF-1
Olink IMMUNE RESPONSE v.3204	OID00937	P16278	GLB1	GLB1
Olink IMMUNE RESPONSE v.3204	OID00941	Q15661	TPSAB1	Tryptase-1
Olink IMMUNE RESPONSE v.3204	OID00943	Q9UHC6	CNTNAP2	CNTNAP2
Olink IMMUNE RESPONSE v.3204	OID00952	Q06830	PRDX1	PRDX1
Olink IMMUNE RESPONSE v.3204	OID00953	P30048	PRDX3	PRDX3
Olink IMMUNE RESPONSE v.3204	OID00954	P09038	FGF2	FGF-2
Olink IMMUNE RESPONSE v.3204	OID00955	P30044	PRDX5	PRDX5
Olink IMMUNE RESPONSE v.3204	OID00956	Q8N608	DPP10	DPP10
Olink IMMUNE RESPONSE v.3204	OID00958	Q14203	DCTN1	DCTN1
Olink IMMUNE RESPONSE v.3204	OID00965	Q8NHJ6	LILRB4	B4
Olink IMMUNE RESPONSE v.3204	OID00967	P08727	KRT19	KRT19
Olink IMMUNE RESPONSE v.3204	OID00968	O43736	ITM2A	ITM2A
Olink IMMUNE RESPONSE v.3204	OID00969	P50135	HNMT	HMT
Olink IMMUNE RESPONSE v.3204	OID00971	Q7Z6M3	MILR1	MILR1
Olink IMMUNE RESPONSE v.3204	OID00982	Q9HCM2	PLXNA4	PLXNA4
Olink IMMUNE RESPONSE v.3204	OID00985	Q07065	CKAP4	CKAP4
Olink IMMUNE RESPONSE v.3204	OID00987	O94992	HEXIM1	HEXIM1
Olink IMMUNE RESPONSE v.3204	OID00988	Q8WXI8	CLEC4D	CLEC4D
Olink IMMUNE RESPONSE v.3204	OID00992	P78310	CXADR	CAR
Olink IMMUNE RESPONSE v.3204	OID00995	Q13241	KLRD1	KLRD1
Olink IMMUNE RESPONSE v.3204	OID00999	P52823	STC1	STC-1
Olink IMMUNE RESPONSE v.3204	OID01001	P58499	FAM3B	FAM3B
Olink IMMUNE RESPONSE v.3204	OID01004	O00273	DFFA	DFFA
Olink IMMUNE RESPONSE v.3204	OID01005	Q96PD2	DCBLD2	DCBLD2
Olink IMMUNE RESPONSE v.3204	OID01009	P15514	AREG	AR
Olink IMMUNE RESPONSE v.3204	OID01010	Q8IU57	IFNLR1	IFN-lambda receptor 1
Olink IMMUNE RESPONSE v.3204	OID01016	Q9BXN2	CLEC7A	CLEC7A
Olink IMMUNE RESPONSE v.3204	OID01018	O95786	RIGI	RIGI
Olink IMMUNE RESPONSE v.3204	OID01025	Q01151	CD83	hCD83
Olink IMMUNE RESPONSE v.3204	OID01027	P78410	BTN3A2	BTN3A2
Olink NEUROLOGY v.8013	OID00287	Q9HAN9	NMNAT1	NMN/NaMN adenylyltransferase 1
Olink NEUROLOGY v.8013	OID00288	O60462	NRP2	NRP2
Olink NEUROLOGY v.8013	OID00292	O95185	UNC5C	UNC5C
Olink NEUROLOGY v.8013	OID00293	Q2TAL6	VWC2	VWC2

Olink NEUROLOGY v.8013	OID00294	Q9Y336	SIGLEC9	Siglec-9
Olink NEUROLOGY v.8013	OID00295	Q08708	CD300C	CLM-6
Olink NEUROLOGY v.8013	OID00296	P15311	EZR	EZR
Olink NEUROLOGY v.8013	OID00297	Q9H3U7	SMOC2	SMOC2
Olink NEUROLOGY v.8013	OID00298	P41271	NBL1	NBL1
Olink NEUROLOGY v.8013	OID00299	P52798	EFNA4	EFNA4
Olink NEUROLOGY v.8013	OID00300	Q14108	SCARB2	SCARB2
Olink NEUROLOGY v.8013	OID00301	O14594	NCAN	NCAN
Olink NEUROLOGY v.8013	OID00302	Q2VWP7	PRTG	PRTG
Olink NEUROLOGY v.8013	OID00303	Q9HCK4	ROBO2	ROBO2
Olink NEUROLOGY v.8013	OID00305	Q96B86	RGMA	RGMA
Olink NEUROLOGY v.8013	OID00306	Q9ULL4	PLXNB3	PLXNB3
Olink NEUROLOGY v.8013	OID00307	P48052	CPA2	CPA2
Olink NEUROLOGY v.8013	OID00308	P28907	CD38	CD38
Olink NEUROLOGY v.8013	OID00309	P17405	SMPD1	SMPD1
Olink NEUROLOGY v.8013	OID00310	P21757	MSR1	MSR1
Olink NEUROLOGY v.8013	OID00311	P30533	LRPAP1	Alpha-2-MRAP
Olink NEUROLOGY v.8013	OID00312	Q92765	FRZB	sFRP-3
Olink NEUROLOGY v.8013	OID00313	O15197	EPHB6	EPHB6
Olink NEUROLOGY v.8013	OID00314	Q6NW40	RGMB	RGMB
Olink NEUROLOGY v.8013	OID00315	Q9BZZ2	SIGLEC1	SIGLEC1
Olink NEUROLOGY v.8013	OID00316	O94779	CNTN5	CNTN5
Olink NEUROLOGY v.8013	OID00317	Q9P0K1	ADAM22	ADAM 22
Olink NEUROLOGY v.8013	OID00318	Q9P126	CLEC1B	CLEC1B
Olink NEUROLOGY v.8013	OID00319	O75077	ADAM23	ADAM 23
Olink NEUROLOGY v.8013	OID00320	O15232	MATN3	MATN3
Olink NEUROLOGY v.8013	OID00321	Q2MKA7	RSPO1	RSPO1
Olink NEUROLOGY v.8013	OID00324	O00214	LGALS8	Gal-8
Olink NEUROLOGY v.8013	OID00325	Q96GW7	BCAN	BCAN
Olink NEUROLOGY v.8013	OID00326	Q6UX15	LAYN	LAYN
Olink NEUROLOGY v.8013	OID00328	O14793	MSTN	GDF-8
Olink NEUROLOGY v.8013	OID00329	P04216	THY1	THY1
Olink NEUROLOGY v.8013	OID00331	Q9H3S3	TMPRSS5	TMPRSS5
Olink NEUROLOGY v.8013	OID00332	P22223	CDH3	CDH3
Olink NEUROLOGY v.8013	OID00333	P56159	GFRA1	GDNF receptor alpha-1
Olink NEUROLOGY v.8013	OID00334	P15509	CSF2RA	GM-CSF-R-alpha
Olink NEUROLOGY v.8013	OID00336	Q6ZMJ2	SCARA5	SCARA5
Olink NEUROLOGY v.8013	OID00337	P41217	CD200	CD200
Olink NEUROLOGY v.8013	OID00338	Q16620	NTRK2	NTRK2
Olink NEUROLOGY v.8013	OID00339	P12544	GZMA	GZMA
Olink NEUROLOGY v.8013	OID00341	Q8NBI3	DRAXIN	DRAXIN
Olink NEUROLOGY v.8013	OID00342	Q96GP6	SCARF2	SCARF2
Olink NEUROLOGY v.8013	OID00343	O60609	GFRA3	GDNF receptor alpha-3
Olink NEUROLOGY v.8013	OID00344	P15151	PVR	PVR
Olink NEUROLOGY v.8013	OID00345	Q9NP84	TNFRSF12A	TNFRSF12A
Olink NEUROLOGY v.8013	OID00346	P37023	ACVRL1	ACVRL1
Olink NEUROLOGY v.8013	OID00347	O43155	FLRT2	FLRT2
Olink NEUROLOGY v.8013	OID00348	P14384	CPM	CPM

Olink NEUROLOGY v.8013	OID00350	P78333	GPC5	GPC5
Olink NEUROLOGY v.8013	OID00351	P12644	BMP4	BMP-4
Olink NEUROLOGY v.8013	OID00353	Q8NFP4	MDGA1	MDGA1
Olink NEUROLOGY v.8013	OID00355	P16234	PDGFRA	PDGF-R-alpha
Olink NEUROLOGY v.8013	OID00356	P53634	CTSC	CTSC
Olink NEUROLOGY v.8013	OID00357	P55285	CDH6	CDH6
Olink NEUROLOGY v.8013	OID00358	Q08345	DDR1	Epithelial discoidin domain receptor 1
Olink NEUROLOGY v.8013	OID00359	P57087	JAM2	JAM-B
Olink NEUROLOGY v.8013	OID00360	P25774	CTSS	CTSS
Olink NEUROLOGY v.8013	OID00361	Q9NR71	ASAH2	N-CDase
Olink NEUROLOGY v.8013	OID00362	Q02083	NAAA	NAAA
Olink NEUROLOGY v.8013	OID00363	Q9BZM5	ULBP2	ULBP2
Olink NEUROLOGY v.8013	OID00365	O75509	TNFRSF21	TNFRSF21
Olink NEUROLOGY v.8013	OID00366	Q8TDQ1	CD300LF	CLM-1
Olink NEUROLOGY v.8013	OID00367	Q08629	SPOCK1	SPOCK1
Olink NEUROLOGY v.8013	OID00368	P29460,P29459		
Olink NEUROLOGY v.8013	OID00369	Q9UBT3	DKK4	Dickkopf-4
Olink NEUROLOGY v.8013	OID00370	Q9HAV5	EDA2R	EDA2R
Olink NEUROLOGY v.8013	OID00372	Q16288	NTRK3	NTRK3
Olink NEUROLOGY v.8013	OID00373	Q6ISS4	LAIR2	LAIR-2
Olink NEUROLOGY v.8013	OID00374	P55145	MANF	MANF
Olink NEUROLOGY v.8013	OID00375	Q92752	TNR	TN-R
Olink NEUROLOGY v.8013	OID00377	Q92823	NRCAM	Nr-CAM
Olink NEUROLOGY v.8013	OID00378	Q16719	KYNU	KYNU
Olink METABOLISM v.3404	OID01120	Q9H6B4	CLMP	CLMP
Olink METABOLISM v.3404	OID01121	Q96JA1	LRIG1	LIG-1
Olink METABOLISM v.3404	OID01122	O95502	NPTXR	NPTXR
Olink METABOLISM v.3404	OID01123	P23526	AHCY	AdoHcyase
Olink METABOLISM v.3404	OID01124	P52888	THOP1	THOP1
Olink METABOLISM v.3404	OID01125	P43234	CTSO	CTSO
Olink METABOLISM v.3404	OID01126	Q96LA6	FCRL1	FcR-like protein 1
Olink METABOLISM v.3404	OID01127	Q04900	CD164	MUC-24
Olink METABOLISM v.3404	OID01128	P20711	DDC	AADC
Olink METABOLISM v.3404	OID01129	Q9NPH0	ACP6	ACP6
Olink METABOLISM v.3404	OID01130	Q03403	TFF2	TFF2
Olink METABOLISM v.3404	OID01132	O15123	ANGPT2	ANG-2
Olink METABOLISM v.3404	OID01133	Q9Y5K6	CD2AP	CD2AP
Olink METABOLISM v.3404	OID01134	O43827	ANGPTL7	ANGPTL7
Olink METABOLISM v.3404	OID01135	Q9NY25	CLEC5A	CLEC5A
Olink METABOLISM v.3404	OID01136	Q9GZM7	TINAGL1	TINAGL1
Olink METABOLISM v.3404	OID01137	P35754	GLRX	GLRX
Olink METABOLISM v.3404	OID01138	P09104	ENO2	ENO2
Olink METABOLISM v.3404	OID01139	O95544	NADK	NADK
Olink METABOLISM v.3404	OID01140	Q9UBU3	GHRL	GHRL
Olink METABOLISM v.3404	OID01141	P50452	SERPINB8	SERPINB8
Olink METABOLISM v.3404	OID01142	P35237	SERPINB6	SERPINB6
Olink METABOLISM v.3404	OID01144	Q76M96	CCDC80	CCDC80
Olink METABOLISM v.3404	OID01146	Q8N1Q1	CA13	CA13

Olink METABOLISM v.3404	OID01147	Q13275	SEMA3F	SEMA3F
Olink METABOLISM v.3404	OID01148	O43240	KLK10	KLK10
Olink METABOLISM v.3404	OID01149	Q9UKJ0	PILRB	PILRB
Olink METABOLISM v.3404	OID01150	O95841	ANGPTL1	ANGPTL1
Olink METABOLISM v.3404	OID01151	P51693	APLP1	APLP1
Olink METABOLISM v.3404	OID01153	P19971	TYMP	TP
Olink METABOLISM v.3404	OID01155	A6NI73	LILRA5	LILRA5
Olink METABOLISM v.3404	OID01156	P00352	ALDH1A1	ALDH1A1
Olink METABOLISM v.3404	OID01157	P40259	CD79B	CD79B
Olink METABOLISM v.3404	OID01160	Q9Y286	SIGLEC7	Siglec-7
Olink METABOLISM v.3404	OID01162	P09417	QDPR	QDPR
Olink METABOLISM v.3404	OID01164	P27695	APEX1	APEX1
Olink METABOLISM v.3404	OID01165	O75356	ENTPD5	ENTPD5
Olink METABOLISM v.3404	OID01166	Q9H4D0	CLSTN2	CLSTN2
Olink METABOLISM v.3404	OID01167	P21964	COMT	COMT
Olink METABOLISM v.3404	OID01168	Q15846	CLUL1	CLUL1
Olink METABOLISM v.3404	OID01169	P51858	HDGF	HDGF
Olink METABOLISM v.3404	OID01170	Q6WN34	CHRDL2	CHRDL2
Olink METABOLISM v.3404	OID01171	P09668	CTSH	CTSH
Olink METABOLISM v.3404	OID01172	Q15155	NOMO1	NOMO1
Olink METABOLISM v.3404	OID01174	Q9BQB4	SOST	SOST
Olink METABOLISM v.3404	OID01175	Q92520	FAM3C	FAM3C
Olink METABOLISM v.3404	OID01176	Q8NBS9	TXNDC5	TXNDC5
Olink METABOLISM v.3404	OID01177	P41236	PPP1R2	IPP-2
Olink METABOLISM v.3404	OID01178	Q9UHL4	DPP7	DPP7
Olink METABOLISM v.3404	OID01179	Q86VZ4	LRP11	LRP-11
Olink METABOLISM v.3404	OID01180	Q9UHX3	ADGRE2	ADGRE2
Olink METABOLISM v.3404	OID01181	Q6UWV6	ENPP7	E-NPP 7
Olink METABOLISM v.3404	OID01182	Q8WTU2	SSC4D	SSC4D
Olink METABOLISM v.3404	OID01183	Q8NI22	MCFD2	MCFD2
Olink METABOLISM v.3404	OID01184	Q9BYZ8	REG4	REG-4
Olink METABOLISM v.3404	OID01185	Q8NBJ7	SUMF2	SUMF2
Olink METABOLISM v.3404	OID01186	Q8WVQ1	CANT1	SCAN-1
Olink METABOLISM v.3404	OID01187	P29017	CD1C	CD1C
Olink METABOLISM v.3404	OID01188	P22466	GAL	GAL
Olink METABOLISM v.3404	OID01189	P19022	CDH2	CDH2
Olink METABOLISM v.3404	OID01190	Q06418	TYRO3	TYRO3
Olink METABOLISM v.3404	OID01191	P46109	CRKL	CRKL
Olink METABOLISM v.3404	OID01192	Q8WX77	IGFBPL1	IGFBPL1
Olink METABOLISM v.3404	OID01193	Q9BZR6	RTN4R	RTN4R
Olink METABOLISM v.3404	OID01194	P13611	VCAN	VCAN
Olink METABOLISM v.3404	OID01195	P09467	FBP1	FBPase 1
Olink METABOLISM v.3404	OID01196	P01222	TSHB	TSHB
Olink METABOLISM v.3404	OID01198	Q92692	NECTIN2	NECTIN2
Olink METABOLISM v.3404	OID01202	P31431	SDC4	SYND4
Olink METABOLISM v.3404	OID01203	Q9NWQ8	PAG1	PAG1
Olink METABOLISM v.3404	OID01204	Q16773	KYAT1	KYAT1
Olink METABOLISM v.3404	OID01206	Q9NQX5	NPDC1	NPDC-1

Olink METABOLISM v.3404	OID01207	Q641Q3	METRNL	METRNL
Olink METABOLISM v.3404	OID01209	Q01973	ROR1	ROR1
Olink METABOLISM v.3404	OID01211	P12724	RNASE3	ECP
Olink ORGAN DAMAGE v.3311	OID01034	P30838	ALDH3A1	ALDH3A1
Olink ORGAN DAMAGE v.3311	OID01039	Q03426	MVK	MK
Olink ORGAN DAMAGE v.3311	OID01040	Q9Y653	ADGRG1	ADGRG1
Olink ORGAN DAMAGE v.3311	OID01041	Q9NQ88	TIGAR	TIGAR
Olink ORGAN DAMAGE v.3311	OID01043	P01588	EPO	EPO
Olink ORGAN DAMAGE v.3311	OID01047	P42658	DPP6	DPP6
Olink ORGAN DAMAGE v.3311	OID01049	Q13145	BAMBI	BAMBI
Olink ORGAN DAMAGE v.3311	OID01050	P40121	CAPG	CAPG
Olink ORGAN DAMAGE v.3311	OID01052	P80303	NUCB2	NUCB2
Olink ORGAN DAMAGE v.3311	OID01058	Q11201	ST3GAL1	Alpha 2,3-ST 1
Olink ORGAN DAMAGE v.3311	OID01061	Q9ULX7	CA14	CA14
Olink ORGAN DAMAGE v.3311	OID01071	Q9Y5L3	ENTPD2	NTPDase 2
Olink ORGAN DAMAGE v.3311	OID01080	Q86SR1	GALNT10	GALNT10
Olink ORGAN DAMAGE v.3311	OID01081	P35070	BTC	BTC
Olink ORGAN DAMAGE v.3311	OID01084	P23582	NPPC	NPPC
Olink ORGAN DAMAGE v.3311	OID01086	Q8IUK5	PLXDC1	PLXDC1
Olink ORGAN DAMAGE v.3311	OID01095	P01258	CALCA	CALCA
Olink ORGAN DAMAGE v.3311	OID01096	P06850	CRH	CRH
Olink ORGAN DAMAGE v.3311	OID01099	P21246	PTN	PTN
Olink ORGAN DAMAGE v.3311	OID01101	O75787	ATP6AP2	ATP6AP2
Olink ORGAN DAMAGE v.3311	OID01102	P19878	NCF2	NCF-2
Olink ORGAN DAMAGE v.3311	OID01108	Q02246	CNTN2	CNTN2
Olink ORGAN DAMAGE v.3311	OID01113	P20472	PVALB	PVALB
Olink ORGAN DAMAGE v.3311	OID01114	Q9UHF1	EGFL7	EGF-like protein 7
Olink ONCOLOGY III v.4001	OID05404	P0DN86	CGB3	CGB3
Olink ONCOLOGY III v.4001	OID05410	P38484	IFNGR2	IFN-gamma receptor 2
Olink ONCOLOGY III v.4001	OID05411	Q9Y5K2	KLK4	KLK4
Olink ONCOLOGY III v.4001	OID05417	P06865	HEXA	HEXA
Olink ONCOLOGY III v.4001	OID05421	Q9H773	DCTPP1	DCTPP1
Olink ONCOLOGY III v.4001	OID05422	Q9H8J5	MANSC1	MANSC1
Olink ONCOLOGY III v.4001	OID05423	P55789	GFER	GFER
Olink ONCOLOGY III v.4001	OID05427	P02745	C1QA	C1QA
Olink ONCOLOGY III v.4001	OID05429	P13521	SCG2	SCG2
Olink ONCOLOGY III v.4001	OID05430	Q9Y644	RFNG	RFNG
Olink ONCOLOGY III v.4001	OID05439	P62166	NCS1	NCS-1
Olink ONCOLOGY III v.4001	OID05451	Q96J42	TXNDC15	TXNDC15
Olink ONCOLOGY III v.4001	OID05452	Q9UMF0	ICAM5	ICAM-5
Olink ONCOLOGY III v.4001	OID05456	Q6PCB0	VWA1	VWA1
Olink ONCOLOGY III v.4001	OID05458	P20849	COL9A1	COL9A1
Olink ONCOLOGY III v.4001	OID05460	Q92832	NELL1	NELL1
Olink ONCOLOGY III v.4001	OID05466	O95715	CXCL14	CXCL14
Olink ONCOLOGY III v.4001	OID05467	O14558	HSPB6	HspB6
Olink ONCOLOGY III v.4001	OID05470	Q53H82	LACTB2	LACTB2
Olink ONCOLOGY III v.4001	OID05474	Q496F6	CD300E	CLM-2
Olink ONCOLOGY III v.4001	OID05475	O60575	SPINK4	SPINK4

Olink ONCOLOGY III v.4001	OID05478	Q9H3G5	CPVL	CPVL
Olink ONCOLOGY III v.4001	OID05487	Q86SF2	GALNT7	GALNT7
Olink ONCOLOGY III v.4001	OID05488	P17948	FLT1	VEGFR-1
Olink ONCOLOGY III v.4001	OID05491	P32004	L1CAM	N-CAM-L1
Olink ONCOLOGY III v.4001	OID05492	Q99795	GPA33	GPA33
Olink ONCOLOGY III v.4001	OID05494	Q08174	PCDH1	PCDH1
Olink ONCOLOGY III v.4001	OID05495	P01303	NPY	NPY
