# Supplementary material for: Aqueous Humor Biomarkers, Efficacy, and Safety in Patients with Naïve Diabetic Macular Edema Treated with Faricimab: The ALTIMETER Study
Source: Ophthalmol Sci. 2026 Feb 26;6(5):101129. doi: 10.1016/j.xops.2026.101129 (PMC13123605; doi:10.1016/j.xops.2026.101129)
Supplement: Table S3 [file mmc9.pdf]

Supplementary Table S3. Exploratory and Post Hoc Endpoints for the Study That Had the Objective to Explore the Associations Over Time Between Clinical Assessments, Multimodal Imaging Assessments, Aqueous Humor Biomarker Patterns, and Genetic Polymorphisms.

- Proportion of patients with a 2-step ETDRS DRSS improvement over time
- Proportion of patients with a 3-step ETDRS DRSS improvement over time
- Changes from baseline on the ETDRS DRSS over time
- Changes from baseline in BCVA (as measured on the ETDRS chart) over time
- Changes from baseline in IRF over time
- Changes from baseline in SRF over time
- Changes from baseline in CST over time
- Changes from baseline in multimodal imaging over time including:
    - FAZ, ischemic index, ischemic area, and MAs (location and number)
    - CST, IRF, SRF, HRF, cyst reflectivity, and DRIL in OCT en-face and volumes
    - Macular leakage over time
- Changes from baseline in AH biomarker patterns over time including:
    - Proteins, including but not limited to: cytokines, chemokines, and growth-, angiogenesis-, and complement-related factors, etc.
    - Metabolites, including but not limited to: lipids, sugars, amino acids, etc.
- Genetic polymorphisms via standard clinical genotyping
- Advanced analytics tools (e.g., artificial intelligence-based tools) for the assessment of clinically relevant features

AH = aqueous humor; BCVA = best-corrected visual acuity; CST = central subfield thickness; DRIL = disorganization of retinal inner layers; DRSS = Diabetic Retinopathy Severity Scale; ETDRS = Early Treatment Diabetic Retinopathy Study; FAZ = foveal avascular zone; HRF = hyperreflective foci; IRF = intraretinal fluid; MA = microaneurysm; OCT = optical coherence tomography; SRF = subretinal fluid.
